# AML‐Targeted Metal‐Polyphenol Nanoplatform Induces Ferroptosis‐ICD Cascade for Antitumor Immunity Boosting

**DOI:** 10.1002/advs.202520544

**Published:** 2026-03-03

**Authors:** Shangqin Yang, Jingxuan Wang, Kerong Tu, Xiaobing Huang, Liangliang Lv, Mingjie Peng, Qiqi Xu, Hongmei Liu, Qiang Sun, Lulu Cai

**Affiliations:** ^1^ Department of Hematology, School of Medicine, Sichuan Provincial People's Hospital University of Electronic Science and Technology of China Chengdu China; ^2^ School of Pharmacy Southwest Medical University Luzhou China; ^3^ Department of Pharmacy Personalized Drug Research and Therapy Key Laboratory of Sichuan Province, Sichuan Provincial People’s Hospital, University of Electronic Science and Technology of China Chengdu China; ^4^ College of Pharmacy Chengdu University Chengdu China

**Keywords:** acute myeloid leukemia, immunosuppression, metal‐polyphenol nanoplatform, shikonin, targeted drug delivery

## Abstract

Acute myeloid leukemia (AML) remains a therapeutic challenge due to its low immunogenicity and immunosuppressive tumor microenvironment (iTME). We developed a ferritin‐based nanoplatform (Fe‐SH@Fn) co‐delivering shikonin (SH) and Fe^3+^ to synergistically induce ferroptosis and activate immunogenic cell death (ICD). Ferritin, a ligand for CD71, which is overexpressed on AML cells, enables tumor targeting and encapsulates Fe‐SH formed by coordination between SH and Fe^3+^. In AML cells, high intracellular glutathione triggers Fe‐SH@Fn disassembly: Fe^3+^ reduces to Fe^2+^ to drive Fenton reaction‐mediated ferroptosis, while SH induces ICD. This dual effect boosts the release of damage‐associated molecular patterns, remodeling the microenvironment by promoting dendritic cell maturation, enhancing CD8^+^ T cell infiltration, and reducing regulatory T cells. In vivo, Fe‐SH@Fn achieved 81.25% tumor growth inhibition in subcutaneous AML model and extended median survival by 2.8‐fold in orthotopic AML model without systemic toxicity. This work presents a translatable strategy that overcomes AML immunosuppression through targeted drug delivery and synergistic ferroptosis‐ICD activation.

## Graphical abstract

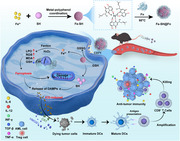

## 1 Introduction

Acute myeloid leukemia (AML) is an aggressive hematologic malignancy characterized by the uncontrolled proliferation of immature myeloid blasts in the peripheral blood, bone marrow, and extramedullary tissues, frequently accompanied by arthralgia, recurrent infections, and anemia [1, 2]. While advances in induction chemotherapy, epigenetic modifiers, and allogeneic hematopoietic stem cell transplantation have improved patient outcomes [3, 4], the clinical management of AML remains hindered by intrinsic heterogeneity, chemoresistance, and treatment‐related toxicities, culminating in high relapse rates [5, 6].

Immunotherapy has emerged as a promising strategy to disrupt the tumor‐immune equilibrium and reignite antitumor immunity through diverse mechanisms [7, 8, 9, 10]. However, current modalities‐including antibody‐drug conjugates and immune checkpoint inhibitors are limited by the inherently low immunogenicity of AML cells and an immunosuppressive tumor microenvironment (iTME), yielding response rates as low as 5%‐30% [11, 12]. Consequently, novel strategies to augment immunogenicity and potentiate immunotherapy efficacy are urgently needed to improve AML treatment paradigms.

Immunogenic cell death (ICD), a specialized form of regulated cell death, elicits adaptive immunity through the release of damage‐associated molecular patterns (DAMPs), including surface‐exposed calreticulin (CRT), extracellular adenosine triphosphate (ATP), and secreted high‐mobility group box 1 (HMGB1) [13, 14]. Upon recognition by pattern recognition receptor (PRR), these DAMPs remodel the iTME by enhancing tumor immunogenicity and recruiting and activation of cytotoxic T lymphocytes [15]. However, conventional ICD inducers (e.g., bleomycin, spautin‐1) suffer from poor tumor selectivity and dose‐limiting systemic toxicity [16, 17]. Moreover, ICD often coincides with immune‐silent apoptotic cell death, which dampens antitumor immune responses [18]. In contrast,​ ferroptosis is an iron‐dependent, lipid peroxidation (LPO)‐driven cell death mode that has emerged as a potent activator of antitumor immunity [19]. Ferroptosis cells release immunostimulatory DAMPs, thereby enhancing tumor immunogenicity and priming T‐cell responses [20, 21]. Thus, developing potent yet low‐toxicity agents capable of inducing immunogenic ferroptosis represents a promising strategy to improve AML immunotherapy.

The natural polyphenol shikonin (SH), an inducer of ICD, has demonstrated broad anticancer activity in solid tumors [22]. SH‐triggered ICD promotes dendritic cell (DC) maturation, antigen cross‐presentation, and T‐cell infiltration via autologous tumor antigen release [23]. However, its immunotherapeutic efficacy in AML remains unexplored, and clinical translation is hindered by poor bioavailability and nonspecific biodistribution. The phenolic hydroxyl groups of SH enable chelation with metal ions (e.g., Fe^3+^), forming stable coordination nanostructures (Fe‐SH) that preserve SH's bioactivity while improving biocompatibility [24, 25, 26, 27, 28]. Critically, in glutathione (GSH)‐rich TME, the Fe^3+^ is reduced to Fe^2+^ and catalyzes the Fenton reaction, amplifying ferroptosis via reactive oxygen species (ROS) generation [29]. Given that AML cells overexpress CD71 (transferrin receptor 1, a poor prognostic marker) [30], we employed ferritin (Fn)‐a natural CD71 ligand with exceptional thermostability as an AML‐targeted delivery vehicle for Fe‐SH coordination nanocomposites. Fn facilitates the self‐assembly of Fe‐SH coordination nanocomposites (Fe‐SH@Fn) through iron‐mediated coordination [31].

**SCHEME 1:**
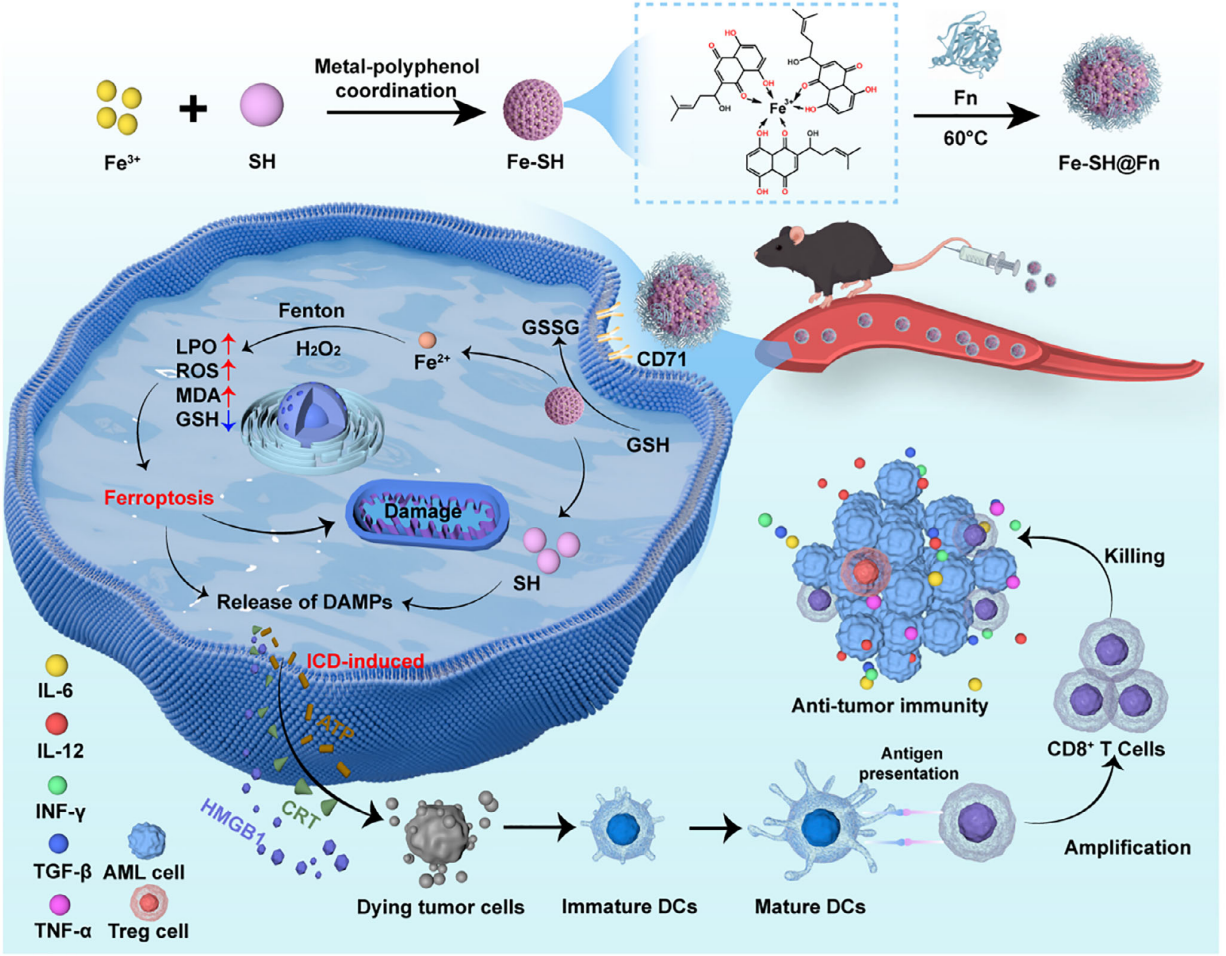
Schematic illustration of the synthesis protocol for Fe‐SH@Fn, an AML‐targeted nanoplatform engineered to co‐deliver shikonin (SH) and Fe^3+^, synergistically boosting antitumor immunity via ferroptosis induction and immunogenic cell death (ICD).

Herein, we designed Fe‐SH@Fn as a supramolecular nanocomposite that integrates: (1) SH‐mediated ICD induction, (2) Fe^2+^‐driven ferroptosis via Fenton reactions, and (3) Fn‐enabled tumor‐targeted delivery. Following cellular uptake by AML cells, Fe‐SH@Fn disassembles in response to the tumor microenvironment (TME), releasing SH and Fe^2+^ to synergistically amplify cytotoxic ferroptosis and ICD. In both orthotopic and subcutaneous AML models, Fe‐SH@Fn achieved significant tumor suppression without detectable systemic toxicity. Collectively, this work establishes an Fn‐based metal‐polyphenol supramolecular self‐assembly strategy that triggers self‐reinforcing immunogenic ferroptosis, overcoming the limitations associated with conventional ICD inducers. By co‐delivering SH and Fe^3+^ with high specificity, Fe‐SH@Fn represents a novel paradigm for enhancing AML immunotherapy (Scheme 1).

## 2 Results and Discussion

### 2.1 Synthesis and Characterization of Fe‐SH@Fn

**FIGURE 1:**
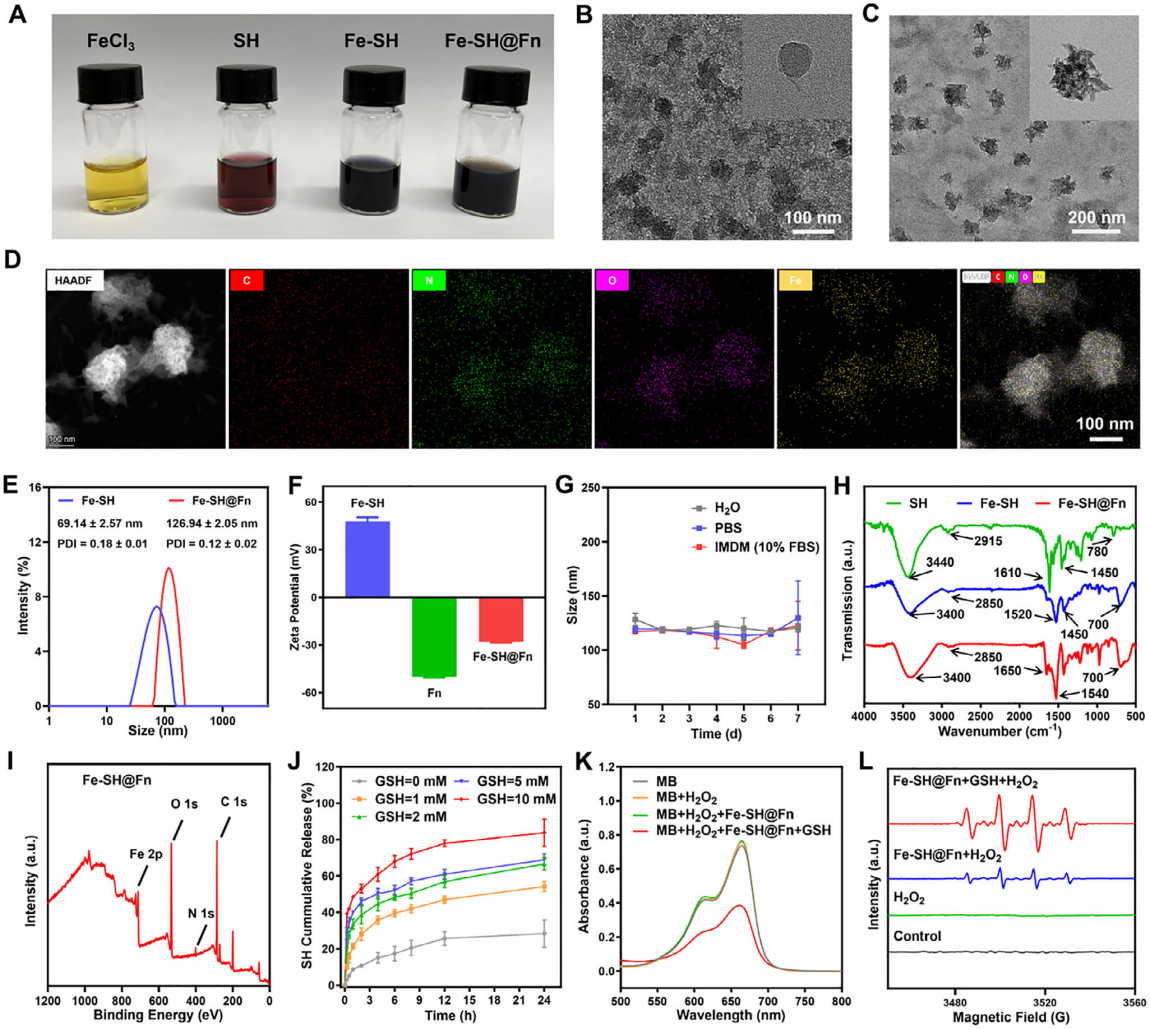
Synthesis and characterization of Fe‐SH and Fe‐SH@Fn nanocomposites. (A) Photographic images of FeCl_3_, SH, Fe‐SH, and Fe‐SH@Fn showing the color evolution during synthesis. (B) TEM micrographs of Fe‐SH nanoparticles. (C) TEM micrographs of Fe‐SH@Fn nanoparticles. (D) EDS elemental mapping of Fe‐SH@Fn, confirming distribution of C, N, O, and Fe. (E) Hydrodynamic size distribution of Fe‐SH and Fe‐SH@Fn. (F) Zeta potential measurements of Fe‐SH, Fn, and Fe‐SH@Fn demonstrating charge‐mediated assembly. (G) Stability assessment of Fe‐SH@Fn under physiological conditions. (H) FTIR spectra revealing characteristic vibrational modes of SH (phenolic ─OH at 3400 cm^−1^), Fe‐SH (metal‐phenolate coordination), and Fe‐SH@Fn (amide at 1650 cm^−1^). (I) XPS full spectrum of Fe‐SH@Fn. (J) GSH‐responsive drug release kinetics of Fe‐SH@Fn. (K) MB degradation assay validating ROS generation under simulated TME conditions (GSH, H_2_O_2_). (L) ESR spectra of •OH generation by Fe‐SH@Fn in the presence of H_2_O_2_ or GSH. Data are presented as mean ± SD (*n* = 3).

The Fe‐SH@Fn was successfully fabricated through a metal‐polyphenol coordination strategy, as schematically illustrated in Scheme 1. Distinct color transitions were observed during the synthesis process (Figure 1A). Transmission electron microscopy (TEM, Figure 1B) and scanning electron microscopy analysis (SEM, Figure S1) demonstrated that Fe‐SH nanoparticles were monodisperse with a uniform spherical morphology. The successful incorporation of Fn was further confirmed by energy‐dispersive X‐ray spectroscopy (EDS) elemental mapping, which showed characteristic signals of carbon (C), nitrogen (N), oxygen (O), and iron (Fe), consistent with the composite composition of Fe‐SH@Fn (Figure 1D). Upon interaction with Fn, Fe‐SH nanoparticles exhibited a pronounced increase in particle size (Figure 1C). Dynamic light scattering (DLS) analysis demonstrated that the hydrated particle size increased from approximately 70 nm for Fe‐SH to ∼120 nm for Fe‐SH@Fn (Figure 1E). Zeta potential measurements suggested electrostatic interactions between negatively charged Fn and positively charged Fe‐SH contributed to nanocomposite formation (Figure 1F). The SH and Fe contents in Fe‐SH@Fn were quantified as 0.28 and 1.07 mg/mL by HPLC and ICP‐OES, respectively (Figures S2 and S3). Notably, Fe‐SH@Fn demonstrated relative stability in water, phosphate buffered saline (PBS), and IMDM medium (10% fetal bovine serum), maintaining consistent hydrated particle size within one week (Figure 1G, Figure S4).

Fourier transform infrared (FTIR) spectroscopic analysis revealed characteristic bands at 3400 cm^−1^ (phenolic O─H stretch), 1420, and 1520 cm^−1^ (aromatic C═C stretch) in Fe‐SH, which due to the formation of coordination bonds (Fe─O) between Fe^3+^ and the O atoms of phenolic ─OH in SH, causing the electron cloud density of O atoms to shift towards the metal center. Finally, the vibration peaks of the aromatic ring skeleton of SH (C═C, C─H) were significantly red shifted from 1610 to 1520 cm^−1^, accompanied by a red shift of the 780 peak to 700 cm^−1^, confirming successful coordination between SH and Fe^3+^. In addition, the Fe‐SH@Fn spectrum clear appearance of amide I band (∼1650 cm^−1^, C═O stretch) and amide II band (∼1540 cm^−^^1^, N─H bend) confirms the Fn incorporation (Figure 1H). Notably, that the characteristic peaks of Fe‐SH nanoparticles (2850 cm^−1^, 700 cm^−1^, C─H) still exist, which remind that Fe‐SH nanoparticles have been integrated into Fe‐SH@Fn. Moreover, UV‐vis spectroscopy reveals that, in contrast to SH and Fe^3+^, the Fe‐SH exhibits a distinct new absorption peak at 610 nm. According to previous results, this change is attributed to the coordination between the phenolic ─OH and Fe^3+^, with the new absorption peak originating from charge transfer transitions from SH to Fe^3+^ (Figure S5). Furthermore, the X‐ray photoelectron spectroscopy (XPS) of Fe‐SH@Fn displays characteristic peaks corresponding to C 1s, N 1s, O 1s, and Fe 2p. Notably, the Fe 2p_1/2_ and 2p_3/2_ peaks are located at 724.18 and 710.78 eV, respectively, and a satellite peak (720 eV) appears on the high‐energy side of the main peak (2p_3/2_ peak), indicating the presence of Fe^3^^+^, which provides the basis for charge transfer between SH and Fe^3+^ (Figure 1I, Figures S6 and S7).

The TME‐responsive behavior of Fe‐SH@Fn was evaluated. Under simulated TME conditions (elevated GSH), Fe‐SH@Fn exhibited GSH‐dependent drug release kinetics, achieving ∼90% cumulative SH release within 24 h at 10 mM GSH (Figure 1J; Figure S8). Fe‐SH@Fn demonstrated potent Fenton reaction activity, as evidenced by methylene blue (MB) degradation, showing effective ROS generation in the presence of GSH/H_2_O_2_ (Figure 1K). Electron spin resonance (ESR) spectroscopy further confirmed hydroxyl radicals (•OH) production, displaying the characteristic 1:2:2:1 quartet signal (Figure 1L). These results demonstrate the dual functionality of Fe‐SH@Fn: (1) stimulus‐triggered SH release mediated by TME, and (2) localized ROS generation through Fenton chemistry. The precisely engineered nanocomposite architecture enables spatiotemporal control of therapeutic activity specifically within TME.

Based on all the evidence obtained, we propose that the formation of Fe‐SH@Fn is governed by a hierarchical assembly process driven by metal‐polyphenol coordination and protein‐nanoparticle interactions. Initially, Fe^3+^ coordinates with the phenolic hydroxyl groups of SH to form dynamic Fe─O coordination bonds, giving rise to an extended metal‐polyphenol network [27]. With increasing coordination density, these networks undergo supramolecular aggregation, ultimately yielding nanoscale Fe‐SH particles, as evidenced by the characteristic color change, UV‐vis absorption at 610 nm, FTIR red shifts, and XPS analysis [32]. Upon the introduction of Fn, the protein predominantly interacts with the surface of Fe‐SH nanoparticles. The increase in hydrated particle size and the shift in zeta potential after Fn incorporation suggest that electrostatic interactions between negatively charged Fn and positively charged Fe‐SH, together with hydrogen bonding and hydrophobic interactions, contribute to the adsorption of Fn onto the nanoparticle surface. Meanwhile, FTIR spectra showing the coexistence of characteristic bands from both Fe‐SH and Fn further support the formation of a composite structure.

### 2.2 In Vitro Antitumor Efficacy of Fe‐SH@Fn

**FIGURE 2:**
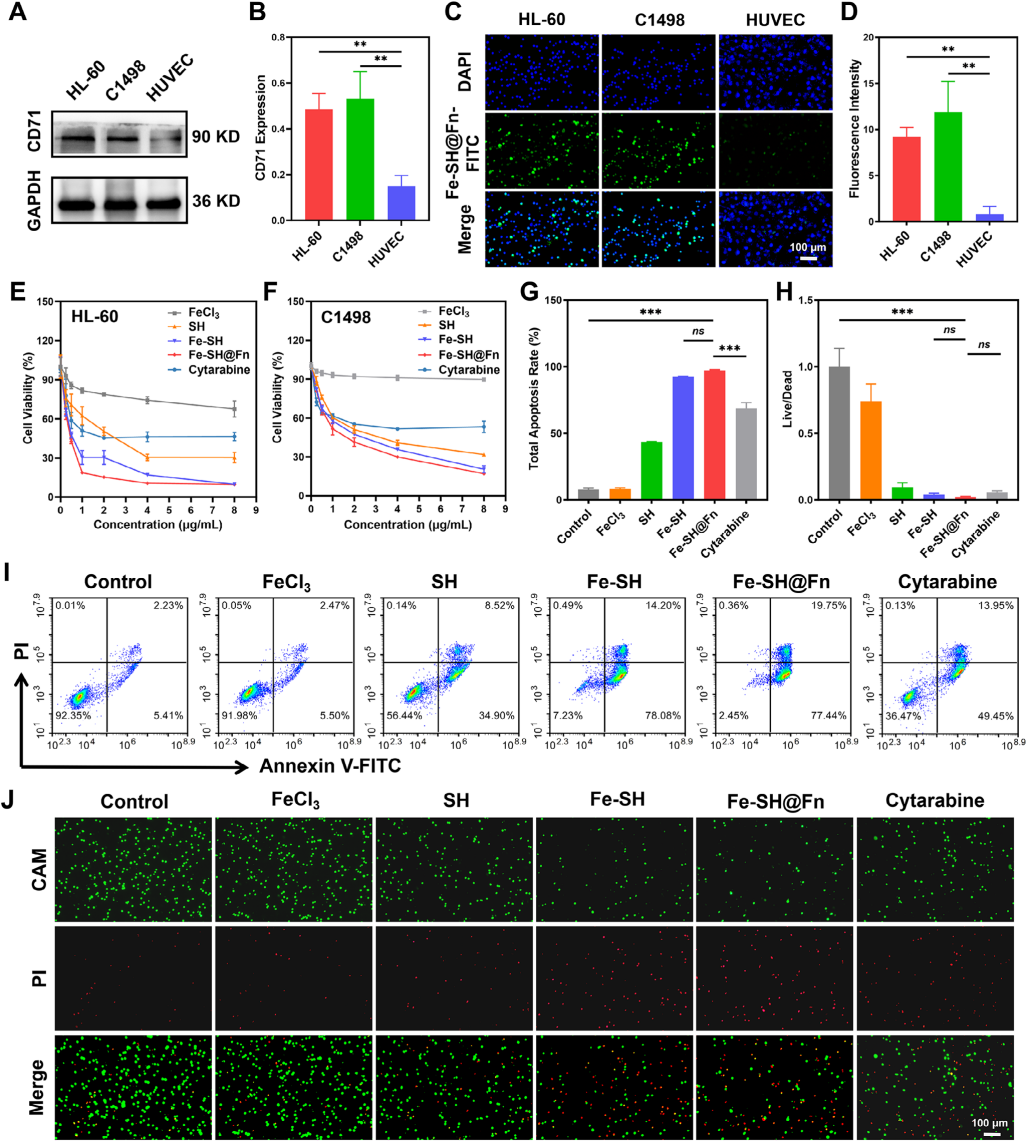
In vitro antitumor evaluation of Fe‐SH@Fn. (A,B) Western blot analysis and quantitative assessment of CD71 expression in HL‐60, C1498, and HUVEC cells. (C,D) Representative fluorescence images and quantitative analysis demonstrating cell‐type specific uptake of Fe‐SH@Fn‐FITC. (E,F) Cell viability profiles of HL‐60 and C1498 cells following various treatments. (G,I) Apoptosis analysis of HL‐60 cells after different treatments by flow cytometry with statistical quantification. (H,J) Live/dead staining and quantitative images of HL‐60 cells after different treatments. Data are presented as mean ± SD (*n* = 3). Statistical significance was assessed using one‐way ANOVA. **p < 0.01, and ***p < 0.001. ns, not significant.

Western blot analysis revealed a distinct expression of transferrin receptor CD71, with high expression observed in AML HL‐60 and C1498 cells, while human umbilical vein endothelial cells (HUVECs) exhibited minimal expression (Figure 2A, B). This differential expression profile endowed Fe‐SH@Fn with inherent selectivity for AML cells. To elucidate the cellular uptake, Fe‐SH@Fn was fluorescently labeled with FITC and evaluated across different cell types. Fluorescence imaging demonstrated significantly enhanced uptake of Fe‐SH@Fn‐FITC in CD71‐positive HL‐60 and C1498 cells after 2 h incubation, whereas minimal uptake was observed in CD71‐deficient HUVECs (Figure 2C,D). Moreover, pre‐treatment with excess Fn to competitively block CD71 resulted in a significant reduction in fluorescence intensity in both HL‐60 and C1498 cells (Figure S9). These results corroborated the CD71‐dependent targeting specificity of our nanoplatform.

The therapeutic efficacy of Fe‐SH@Fn was systematically evaluated through comprehensive in vitro assays. Cell counting kit‐8 (CCK‐8) assays showed that Fe‐SH@Fn induced cell death in HL‐60 and C1498 cells at 24 h (Figure 2E,F), with IC_50_ values of 0.38 and 1.26 µg/mL, respectively. Fe‐SH@Fn showed a significantly enhanced cytotoxicity compared with Fe‐SH (IC_50_ values of 0.57 and 1.6 µg/mL, respectively) and SH (IC_50_ values of 1.75 and 2.19 µg/mL, respectively), which attributable to the enhanced cellular uptake mediated by the Fn‐based delivery system. Flow cytometric analysis of apoptosis revealed that Fe‐SH@Fn induced pronounced early and late apoptotic cell death, resulting in a total apoptosis rate of 97.19%, which was significantly higher than that of the control group (7.64%) (Figure 2G,I, Figure S10). Live/dead staining assays provided visual evidence of the superior cytotoxic effects exerted by Fe‐SH@Fn (Figure 2H,J), which exceeded that of the clinical agent cytarabine. Collectively, these multimodal analyses provide compelling evidence for the anti‐AML activity of Fe‐SH@Fn through CD71‐targeted drug delivery in vitro.

### 2.3 Fe‐SH@Fn Induces Ferroptosis in AML Cells

**FIGURE 3:**
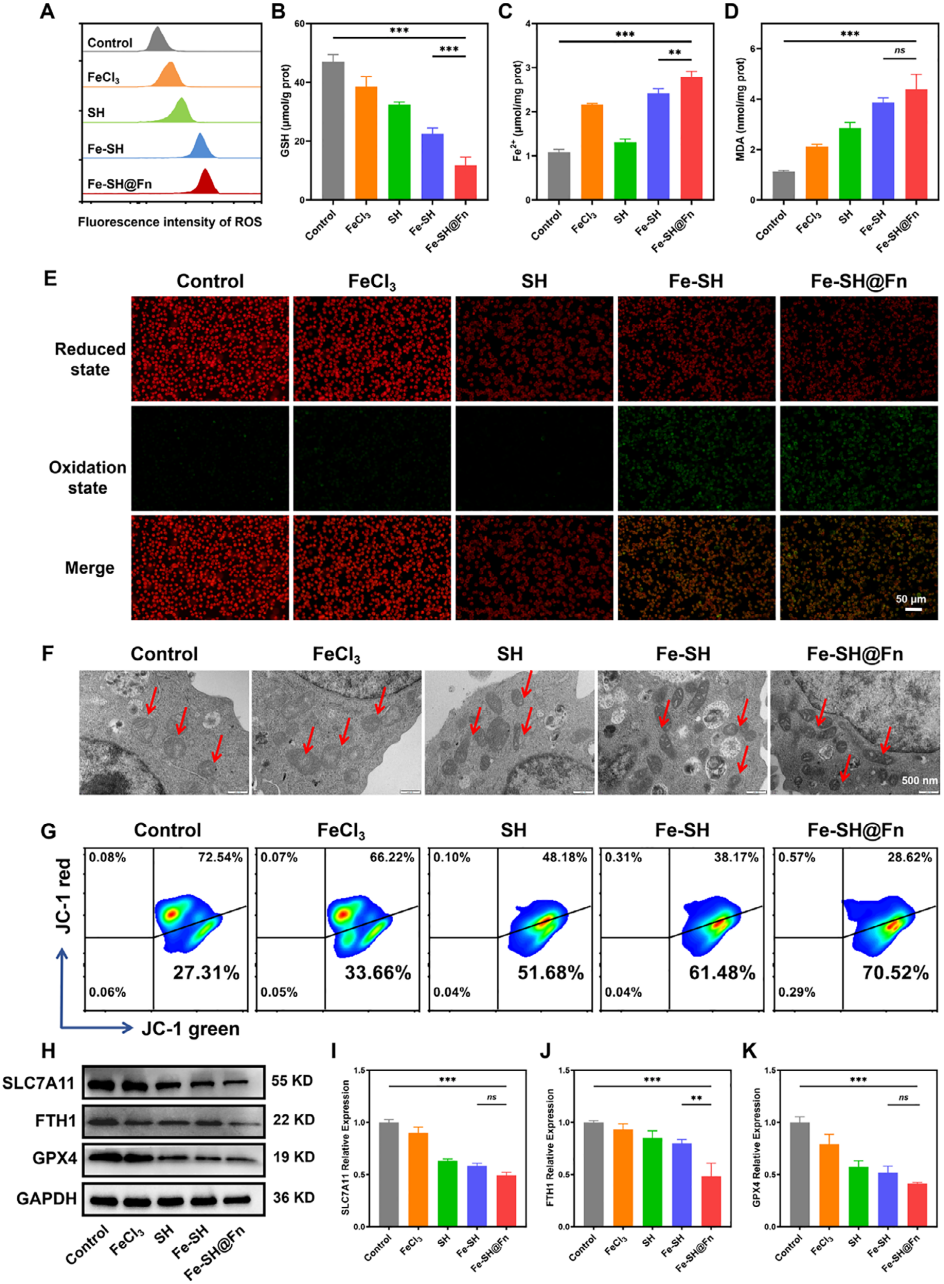
Ferroptosis induction by Fe‐SH@Fn in HL‐60 cells. (A) Flow cytometry analysis of ROS production in HL‐60 cells after treatments of FeCl_3_, SH, Fe‐SH, and Fe‐SH@Fn, respectively. (B–D) Biochemical assays of GSH, Fe^2+^, and MDA levels in HL‐60 cells after different treatments. (E) LPO visualization in HL‐60 cells after different treatments. (F) Bio‐TEM images of mitochondria (Red Arrows) in HL‐60 cells after different treatments. (G) MMP analysis via JC‐1 staining of HL‐60 cells after different treatments. (H–K) Western blot and quantitative analysis of ferroptosis‐related protein (SLC7A11, FTH1, and GPX4) expression. Data are presented as mean ± SD (*n* = 3). Statistical significance was assessed using one‐way ANOVA. **p < 0.01, and ***p < 0.001. ns, not significant.

Ferroptosis, an iron‐dependent form of regulated cell death characterized by LPO, is mechanistically driven by GSH depletion and glutathione peroxidase 4 (GPX4) inactivation [33, 34, 35]. To investigate Fe‐SH@Fn‐induced ferroptosis, we systematically analyzed key ferroptotic markers in HL‐60 cells. Fe‐SH@Fn markedly elevated intracellular ROS, as determined by the DCFH‐DA assay (Figure 3A, Figures S11 and S12). This enhanced oxidative stress was accompanied by significant GSH depletion (Figure 3B) and Fe^2+^ accumulation (Figure 3C). Malondialdehyde (MDA) levels increased by 3.2‐fold versus controls (Figure 3D), confirming oxidative damage. The evidence of LPO was further obtained using C11‐BODIPY581/591 staining, which revealed a pronounced ferroptotic signature in Fe‐SH@Fn group with 82% increase in oxidized (green) fluorescence and 67% reduction in non‐oxidized (red) signal (Figure 3E, Figure S13).

At the subcellular level, biological transmission electron microscopy (Bio‐TEM) revealed characteristic ferroptotic ultrastructural changes, including mitochondrial swelling and cristae fragmentation, following incubation with Fe‐SH and Fe‐SH@Fn (Figure 3F). Consistently, JC‐1 staining demonstrated a significant decrease in mitochondrial membrane potential (MMP) in Fe‐SH@Fn‐treated cells, with a 43% decrease relative to the control group (Figure 3G, Figure S14), indicating substantial mitochondria damage. Mechanistically, Western blot and Immunofluorescence (IF) revealed coordinated downregulation of the key ferroptosis‐related proteins [29, 36, 37], including the cystine/glutamate antiporter (SLC7A11), ferritin heavy chain 1 (FTH1), and the lipid repair enzyme (GPX4). Specifically, the expression levels of SLC7A11, FTH1, and GPX4 were reduced by 51%, 52%, and 59%, respectively, compared with control cells (Figure 3H–K, Figures S15–S17). Collectively, these findings position Fe‐SH@Fn as a potent inducer of iron‐dependent cell death. By simultaneously targeting multiple regulatory nodes of the ferroptosis pathway to exerts a synergistic antileukemic effect that surpasses conventional single‐mechanism therapies. This multimodal mechanism not only enhances efficacy against AML cells but also reduces the likelihood of resistance development, offering promising clinical translation potential for refractory leukemia cases.

### 2.4 Fe‐SH@Fn Induces ICD in AML Cells

**FIGURE 4:**
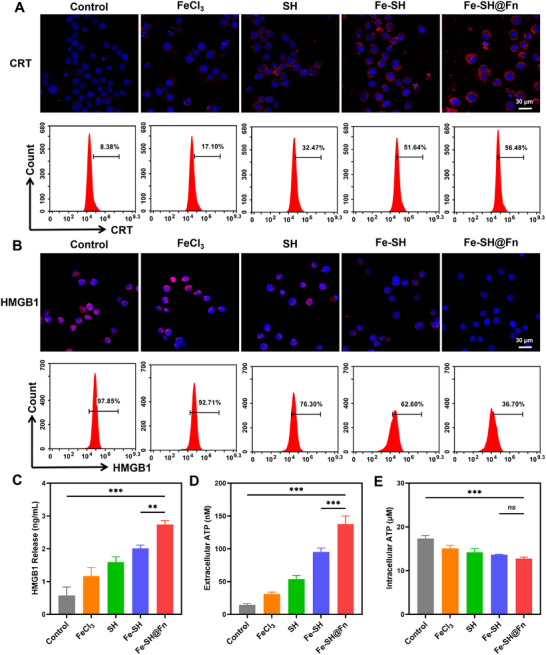
ICD induction in AML cells following treatment with FeCl_3_, SH, Fe‐SH, and Fe‐SH@Fn, respectively. (A) Immunofluorescence and flow cytometric analysis of CRT surface exposure in HL‐60 cells. (B) Immunofluorescence and flow cytometry quantification of HMGB1 release. (C) ELISA‐based measurement of extracellular HMGB1 concentration. (D,E) Luminescent ATP assays quantifying intracellular depletion and extracellular accumulation of ATP. Data are presented as mean ± SD (*n* = 3). Statistical significance was assessed using one‐way ANOVA. **p < 0.01, and ***p < 0.001. ns, not significant.

Building upon recent evidence that ferroptosis enhances the immunogenicity of AML cells through the release of immunostimulatory signals, and SH has been shown to induce ICD [38, 39], we systematically evaluated Fe‐SH@Fn's ability to trigger ICD in vitro [40]. Immunofluorescence and flow cytometric analyses demonstrated a pronounced increase in calreticulin (CRT) exposure on the surface of AML cells following Fe‐SH@Fn treatment, with a 6.7‐fold elevation compared to control cells (Figure 4A, Figure S18). Consistently, HMGB1 exhibited a marked reduction of fluorescence signal within the nucleus (Figure 4B, Figure S19), with ELISA quantification confirming a 2.6‐fold elevation in extracellular HMGB1 release (Figure 4C). Moreover, Fe‐SH@Fn simultaneously depleted intracellular ATP stores while increasing extracellular ATP levels by 3.7‐fold (Figure 4D,E), collectively demonstrating that Fe‐SH@Fn not only induces ferroptosis but also potently amplifies the ICD cascade, thereby enhancing AML cell immunogenicity and potentially bridging innate and adaptive antitumor immunity.

### 2.5 Biodistribution and Antitumor Efficacy of Fe‐SH@Fn In Vivo

**FIGURE 5:**
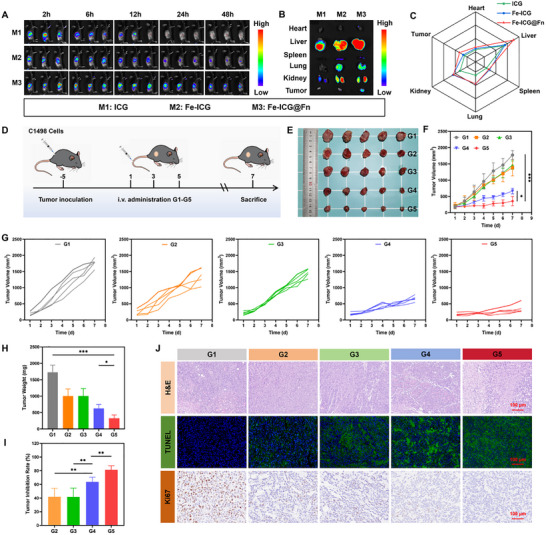
Biodistribution and antitumor efficacy of Fe‐SH@Fn in AML subcutaneous mice. (A) Whole‐body fluorescence imaging showing time‐dependent tumor accumulation (2–48 h) of ICG, Fe‐ICG, and Fe‐ICG@Fn formulations. (B,C) Ex vivo fluorescence distribution in major organs (heart, liver, spleen, lung, kidney) and tumors 6 h post‐injection. (D) Experimental timeline for therapeutic evaluation in C1498 tumor‐bearing C57BL/6 mice. (E) Digital photos of the anatomical tumor tissues after 7 days of treatment. (F, G) Average tumor growth profiles in different groups. (H) Average tumor weight after different treatments. (I) Average tumor inhibition rate after different treatments. (J) H&E, TUNEL (green), and Ki67 (red) staining of tumor tissues after different treatments. Data are presented as mean ± SD (*n* = 5). Statistical significance was assessed using one‐way ANOVA and an unpaired t‐test. *p < 0.05, **p < 0.01, and ***p < 0.001. G1: Control, G2: FeCl_3_, G3: SH, G4: Fe‐SH, G5: Fe‐SH@Fn.

To evaluate the in vivo tumor‐targeting capability of the nanoplatform, tumor‐bearing mice (tumor volume ∼100 mm^3^) were intravenously injected with free ICG, Fe‐ICG, or Fe‐ICG@Fn for real‐time fluorescence imaging. Fe‐ICG@Fn exhibited rapid and pronounced tumor accumulation, with detectable fluorescence as early as 2 h post‐injection and a peak signal at 6 h, which was approximately 1.6‐fold higher than that of free ICG. Notably, strong tumor retention of Fe‐ICG@Fn persisted for up to 24 h, whereas free ICG and Fe‐ICG were rapidly cleared from the tumor site within 2 h (Figure 5A, Figure S20). Ex vivo organ imaging at 6 h confirmed Fn‐mediated tumor targeting, with Fe‐ICG@Fn exhibiting 3.2‐fold higher tumor fluorescence intensity compared to other groups (Figure 5B,C).

The antitumor efficacy of Fe‐SH@Fn was systematically evaluated in C1498 subcutaneous tumor‐bearing C57BL/6 mice, with treatments initiated 7 days post‐inoculation and administered every 3 days (Figure 5D). Fe‐SH@Fn treatment resulted in remarkable tumor growth inhibition (TGI = 81.25%), significantly outperforming other groups (Figure 5E–I), while maintaining excellent systemic safety as evidenced by stable body weight (Figure S21). Histopathological analysis revealed extensive tumor cell necrosis in H&E‐stained sections from Fe‐SH@Fn‐treated mice, with TUNEL assays showing the most apoptosis cells. In addition, immunohistochemical analysis demonstrated a significant reduction in Ki67‐positive proliferating cells in tumor tissues following Fe‐SH@Fn treatment (Figure 5J), indicating effective suppression of tumor proliferation.

Furthermore, Fe‐SH@Fn effectively mitigated AML‐associated splenomegaly, restoring spleen size to near‐normal dimensions (Figure S22), which correlated with its superior tumor‐suppressive effects and confirmed the platform's ability to control systemic disease progression. These comprehensive in vivo results demonstrate that Fe‐SH@Fn combines excellent tumor‐targeting capability with potent therapeutic efficacy against AML while maintaining a favorable safety profile.

### 2.6 Induction of Ferroptosis and ICD by Fe‐SH@Fn In Vivo

**FIGURE 6:**
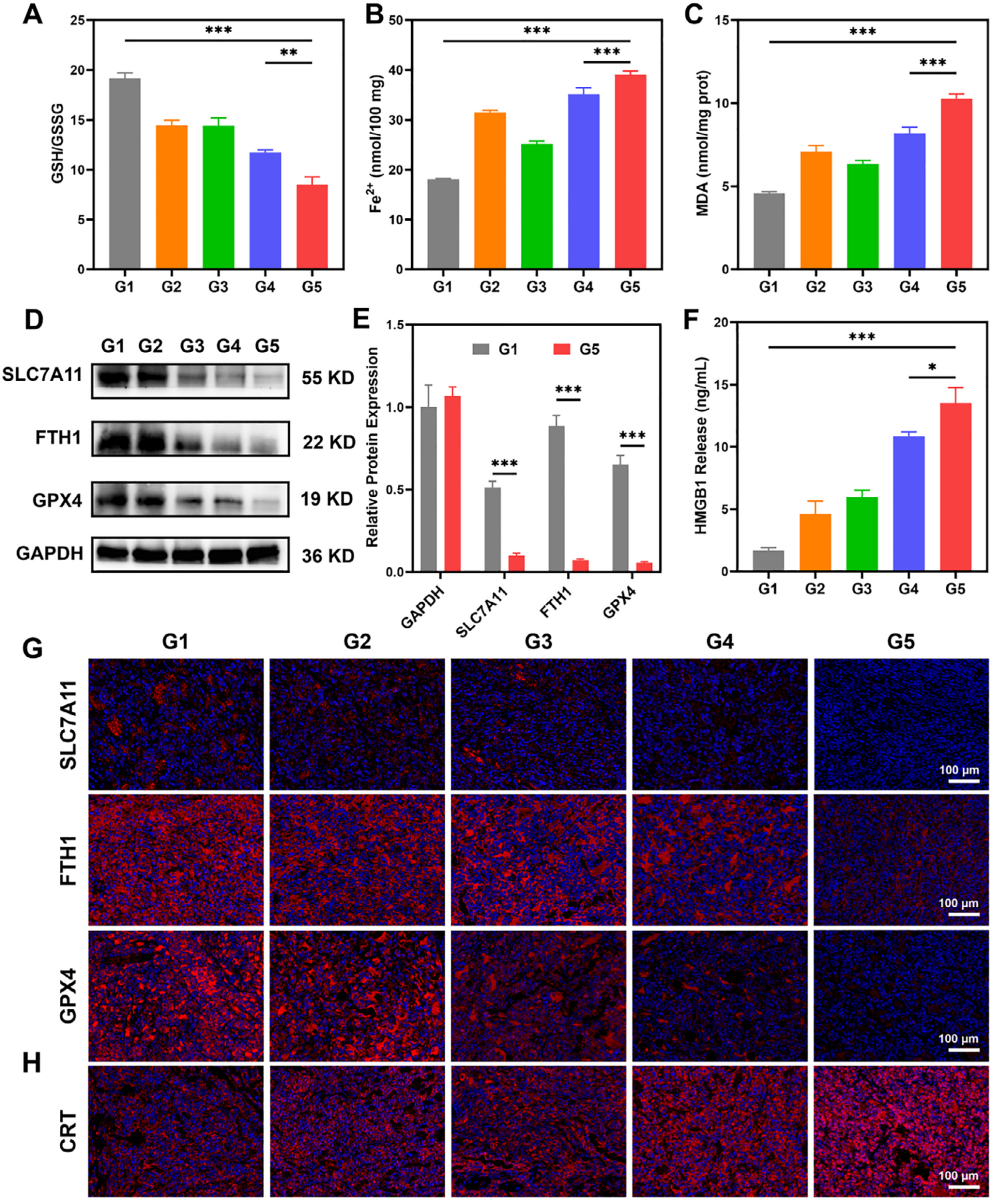
Ferroptosis induction and ICD after different treatments in vivo. (A) Redox imbalance evidenced by decreased GSH/GSSG ratio in tumor tissues. (B) Iron overload is evidenced by intratumoral Fe^2+^ accumulation. (C) LPO is evidenced by elevated intratumoral MDA levels. (D,E) Western blot analysis and quantification showing significant downregulation of ferroptosis regulators SLC7A11, FTH1, and GPX4 in tumor tissues. (F) ELISA quantification of extracellular HMGB1 release. (G,H) Immunofluorescence staining revealed decreased expression of ferroptosis‐related proteins (SLC7A11, FTH1, GPX4) and increased CRT exposure in tumor sections. Data are presented as mean ± SD (*n* = 3). Statistical significance was assessed using one‐way ANOVA and an unpaired *t*‐test. (E) *p < 0.05, **p < 0.01, and ***p < 0.001. G1: Control, G2: FeCl_3_, G3: SH, G4: Fe‐SH, G5: Fe‐SH@Fn.

We systematically evaluated the dual capacity of Fe‐SH@Fn to induce ferroptosis and ICD in the C1498 subcutaneous tumor model. Biochemical analysis of tumor tissues revealed that Fe‐SH@Fn treatment significantly altered key ferroptotic markers, inducing a 2.3‐fold increase in LPO products MDA and 6.5‐fold elevation in Fe^2+^ levels, while depleting GSH by 56% compared to saline controls (Figure 6A–C). Immunofluorescence and western blot analysis demonstrated a substantial downregulation of key ferroptosis‐regulatory proteins, The expression levels of SLC7A11, ferritin heavy chain (FTH1), and glutathione peroxidase 4 (GPX4) were reduced by 80.0%, 90.4%, and 90.0%, respectively (Figure 6D,E,G, Figures S23–S25), confirming that Fe‐SH@Fn effectively activates iron‐dependent lipid peroxidation pathways within the tumor microenvironment.

Parallel evaluation of ICD markers showed that Fe‐SH@Fn treatment triggered robust immunogenic responses. ELISA analysis revealed that Fe‐SH@Fn treatment increased HMGB1 release by 7.9‐fold (Figure 6F) and immunofluorescence demonstrating significant CRT surface exposure (Figure 6H, Figure S26). These findings collectively demonstrate that Fe‐SH@Fn maintains its dual mechanism of action in vivo, simultaneously executing direct tumoricidal effects through ferroptosis while priming antitumor immunity via ICD induction.

### 2.7 Immunomodulatory Effects of Fe‐SH@Fn In Vivo

Subsequently, the immunostimulatory properties of Fe‐SH@Fn were systematically evaluated through comprehensive immune profiling. Flow cytometric analysis showed that Fe‐SH@Fn increased the proportion of mature dendritic cells in tumor‐draining lymph nodes (TDLNs) by 2.8‐fold compared with control groups (Figure S27), directly correlating with its robust DAMPs release profile. This enhanced DC activation facilitated efficient antigen presentation to naive T cells [41, 42], as evidenced by the marked expansion of both CD4^+^ (4.9‐fold increase) and CD8^+^ (5.1‐fold increase) T cell populations in splenocyte analyses (Figure S28), while simultaneously reducing immunosuppressive Tregs by 40% (Figure S29). These findings establish Fe‐SH@Fn's unique bidirectional immunomodulatory capacity, simultaneously amplifying cytotoxic immune responses while attenuating immunosuppressive pathways.

**FIGURE 7:**
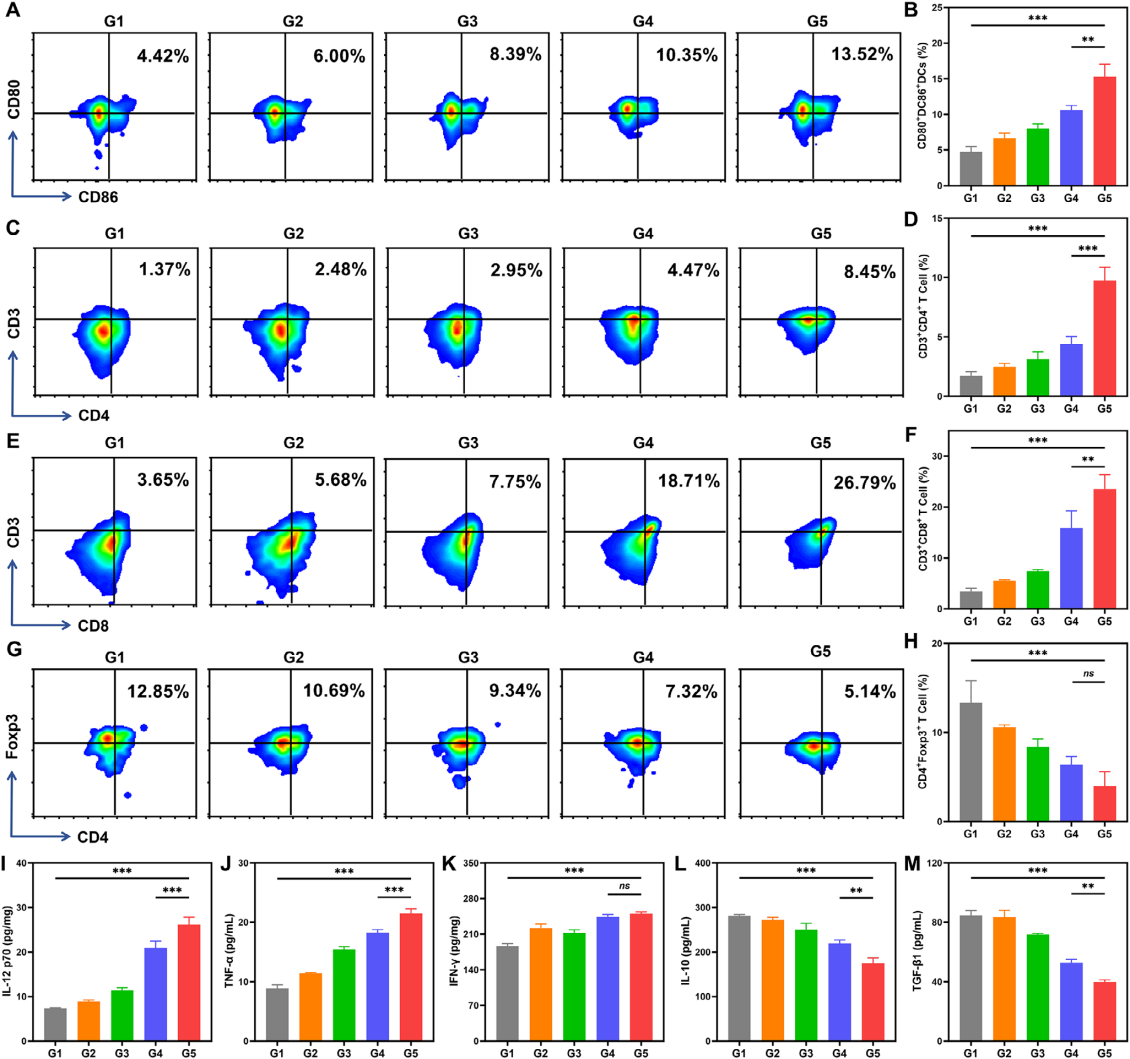
Systemic immune activation and TME remodeling by Fe‐SH@Fn. (A,B) Flow cytometry analysis of mature DCs (CD80^+^CD86^+^) in tumor tissues. (C,D) Flow cytometry analysis of tumor‐infiltrating CD4^+^ T lymphocytes (CD3^+^CD4^+^) in tumor tissues. (E,F) Flow cytometry analysis of cytotoxic CD8^+^ T cell populations (CD3^+^CD8^+^) in tumor tissues. (G,H) Flow cytometry analysis of regulatory T cell (CD4^+^Foxp3^+^) in tumor tissues. (I–M) Serum (TNF‐α, IL‐10, TGF‐β1) and tumor tissue (IL‐12p70, IFN‐γ) cytokine profiles showing pro‐inflammatory and immunosuppressive cytokine modulation by ELISA. Data are presented as mean ± SD (*n* = 3). Statistical significance was assessed using one‐way ANOVA. **p < 0.01, and ***p < 0.001. ns, not significant. G1: Control, G2: FeCl_3_, G3: SH, G4: Fe‐SH, G5: Fe‐SH@Fn.

Detailed characterization of tumor‐infiltrating immune cells provided further mechanistic insights into immunotherapeutic effects of Fe‐SH@Fn. Flow cytometric analysis of tumor tissues revealed a higher proportion of mature dendritic cells in the Fe‐SH@Fn‐treated group (13.52%), compared with control groups (Figure 7A,B), which directly correlated with enhanced T cell infiltration. Quantitative analysis revealed substantial increases in tumor‐infiltrating lymphocytes, with CD4^+^ T cells reaching 8.45% and CD8^+^ T cells achieving 26.79% penetration (Figure 7C–F), while Treg populations were markedly reduced to minimal levels (Figure 7G,H). This immune profile further confirms that Fe‐SH@Fn remodels the TME by promoting effector cell recruitment/activation and suppressing regulatory T cells.

Local and systemic immune activation induced by Fe‐SH@Fn was further validated through cytokine profiling [43, 44]. Compared with control groups, Fe‐SH@Fn treatment significantly elevated intratumoral IL‐12p70 (3.6‐fold) and IFN‐γ levels (1.3‐fold), indicating enhanced local antigen presentation and T cell‐mediated immune activation. Serum levels of the pro‐inflammatory factor TNF‐α increased by 2.4‐fold, while the levels of immunosuppressive cytokines IL‐10 and TGF‐β1 decreased by 38% and 53%, respectively, revealing a significant systemic immune regulatory effect (Figure 7I,M). Collectively, these coordinated immunomodulatory effects indicate that Fe‐SH@Fn reprograms the immunosuppressive tumor microenvironment by promoting DC‐mediated antigen presentation, enhancing cytotoxic T cell responses, and disrupting immunosuppressive networks, thereby establishing a favorable milieu for effective tumor immune surveillance and clearance.

### 2.8 Therapeutic Efficacy in Orthotopic AML Model and Biosafety Assessment of Fe‐SH@Fn

**FIGURE 8:**
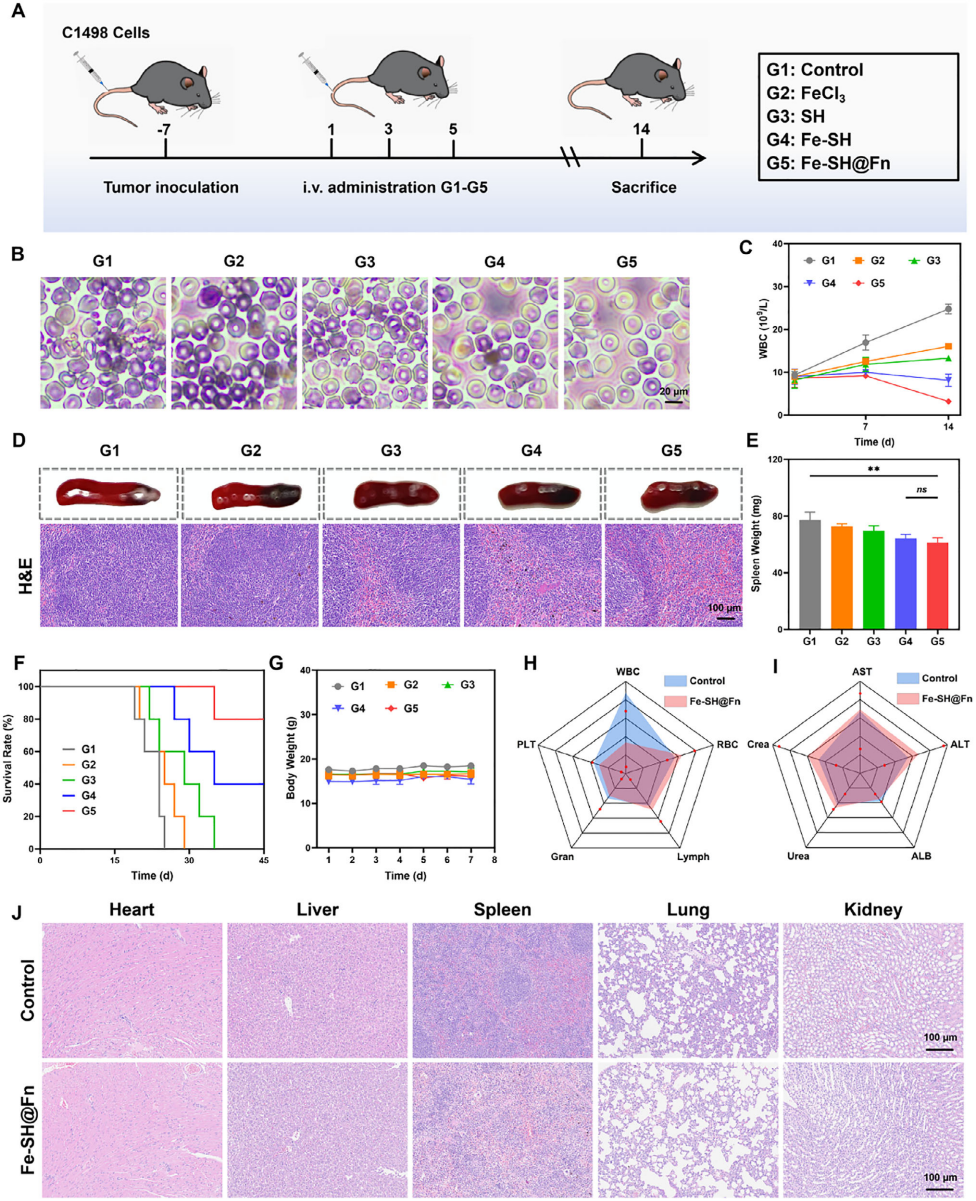
Therapeutic efficacy of Fe‐SH@Fn in the AML orthotopic model.​(A) Experimental timeline for treatment administration in leukemia‐bearing mice. (B) Representative images of Wright‐Giemsa‐stained blood cells. (C) WBC counts in leukemia‐bearing mice. (D) Morphology and H&E staining analysis of spleen. (E) Splenomegaly assessment through organ weight measurement. (F) Survival curves of orthotopic AML mice. (G) Body weight changes of orthotopic AML mice during treatment. (H,I) Blood routine and biochemical indicators of mice after different treatments, the normal range is between two red dots. (J) H&E staining revealed intact tissue architecture without pathological alterations. Data are presented as mean ± SD (*n* = 3). Statistical significance was assessed using one‐way ANOVA. **p < 0.01. ns, not significant. G1: Control, G2: FeCl_3_, G3: SH, G4: Fe‐SH, G5: Fe‐SH@Fn.

To evaluate the systemic therapeutic potential of Fe‐SH@Fn, we established a disseminated AML model in C57BL/6 mice through intravenous injection of C1498 cells. Seven days after inoculation, mice were randomized into five treatment groups that received intravenous administrations every 3 days (Figure 8A). Hematological analysis revealed that Fe‐SH@Fn treatment significantly reduced blood circulating leukemic blasts by 78% compared to untreated controls (Figure 8B,C). Histopathological examination further revealed preservation of normal splenic architecture in Fe‐SH@Fn‐treated mice, whereas control animals exhibited extensive leukemic infiltration and severe structural disruption of the spleen (Figure 8D,E). Importantly, Fe‐SH@Fn prolonged median survival by 2.8‐fold versus saline controls (Figure 8F), demonstrating its robust therapeutic efficacy against systemic disease.

Comprehensive immunophenotyping was performed to investigate the mechanisms underlying Fe‐SH@Fn's therapeutic activity. Analysis of Peripheral blood revealed a 1.7‐fold increase in mature DC populations (Figure S30), accompanied by significant expansion of both CD4^+^ (2.1‐fold) and CD8^+^ (2.0‐fold) T cell subsets (Figure S31). Notably, Fe‐SH@Fn treatment reduced blood circulating Treg ratio by 39.0% compared to controls (Figure S32), indicating effective alleviation of immunosuppression. Collectively, these findings demonstrate that Fe‐SH@Fn established systemic immunomodulatory effects by inducing DC maturation, expanding effector T cell populations, and suppressing Tregs, thereby orchestrating antitumor effects in the AML orthotopic model.

Finally, a comprehensive in vivo biosafety evaluation of Fe‐SH@Fn was conducted through multiparametric analysis, including hematological, biochemical, and histopathological assessments, to systematically assess its safety profile in murine models. Throughout the treatment course, all experimental groups maintained stable body weight trajectories, with no observable signs of distress (Figure 8G). Histopathological examination of major organs (heart, liver, spleen, lung, and kidney) via H&E staining revealed intact tissue architecture without pathological alterations (Figure 8J), confirming good biocompatibility. Hematological monitoring over 14 days post‐administration demonstrated within normal ranges variation in critical blood parameters: white blood cells (WBC), red blood cells (RBC), lymphocytes (Lymph), granulocytes (Gran), and platelets (PLT) (Figure 8H). Hepatic function analysis showed transient alanine aminotransferase elevation (ALT) but within physiological ranges, while aspartate aminotransferase levels remained consistently within physiological ranges (AST). Renal function markers exhibited remarkable stability, with urea and creatinine (Crea) concentrations showing no statistically significant differences (Figure 8I). This comprehensive safety profile, encompassing physiological, histological, hematological, hepatic and renal parameters, establishes Fe‐SH@Fn as a highly biocompatible nanotherapeutic platform with favorable clinical translation potential for AML treatment.

## 3 Conclusion

This study presents an innovative targeted nanotherapeutic platform (Fe‐SH@Fn) that synergistically integrates transferrin receptor‐mediated active targeting with metal‐polyphenol coordination for precision AML therapy. The platform capitalizes on tumor microenvironment‐responsive release of Fe^2+^ and therapeutic drugs to simultaneously induce ferroptosis and amplify ICD, creating a self‐amplifying therapeutic cascade. Fe‐SH@Fn bridges targeted chemotherapy, ferroptosis, and immunotherapy paradigms, achieving potent antitumor immunity with exemplary biosafety. Future study should focus on developing CD71 expression‐based patient stratification strategies, combining CD71‐targeted therapies with conventional AML chemotherapeutics or immune checkpoint inhibitors, and validating efficacy in clinically representative models like patient‐derived xenografts to bridge the translational gap. In summary, this multimodal approach represents a significant conceptual and technical advance in leukemia treatment, offering an AML‐targeted multi‐mechanism treatment strategy that addresses both tumor cell elimination and immune microenvironment remodeling.

## 4 Experimental Section

### 4.1 Materials

Shikonin (SH), FeCl_3_·6H_2_O, horse spleen ferritin (Fn), methylene blue trihydrate (MB), and H_2_O_2_ solution (w/w: 30%) were purchased from Sinopharm Chemical Reagent Co., Ltd. (China). Cell counting kit‐8 (CCK‐8), H_2_O_2_ assay kit, Annexin V‐FITC/PI cell apoptosis kit, BODIPY 581/591 C11, mitochondrial membrane potential assay kit with JC‐1, and 2′,7′‐dichlorodihydrofluorescein diacetate (DCFH‐DA) were purchased from Beyotime Institute of Biotechnology (China). Fetal bovine serum (FBS), RPMI 1640, Iscove's Modified Dulbecco Medium (IMDM) and Dulbecco's modified eagle medium (DMEM) were purchased from Gibco Invitrogen Corp (USA). The following antibodies and reagents were purchased from BD Biosciences (USA): Fixable viability stain 620, APC‐anti‐CD45, FITC‐anti‐CD3, PE‐anti‐CD4, Percp‐anti‐CD8, PE‐anti‐Foxp3, APC‐anti‐CD11c, PE‐anti‐CD86, PE‐anti‐CD80, and Mouse BD Fc Block.

### 4.2 Preparation of Fe‐SH

Fe‐SH nanoparticles were synthesized using one‐pot assembly. Briefly, FeCl_3_·6H_2_O (108 mg) was dissolved in 20 mL of ultrapure water and mixed with an ethanolic solution of SH (7.2 mg/mL) under continuous stirring at room temperature for 90 min. The reaction mixture was purified by high‐speed centrifugation at 12000 rpm for 30 min. The resulting Fe‐SH nanoparticles were dialyzed against ultrapure water using a 200 Da dialysis bag for 48 h and subsequently freeze‐dried. The lyophilized product was stored at −20°C for further use.

### 4.3 Preparation of Fe‐SH@Fn

Briefly Fe‐SH@Fn was prepared by loading Fe‐SH into Fn. Specifically, a solution of Fe‐SH was incubated with a solution of Fn at a molar ratio of 500:1 at 60°C for 4 h. The resulting Fe‐SH@Fn nanoparticles were then dialyzed using a 3500 Da molecular weight cutoff dialysis bag with ultrapure water as the dialysate for 48 h. Finally, the purified product was stored at 4°C.

### 4.4 Characterization of Fe‐SH@Fn

First, DLS was performed to measure particle size, polymer dispersity index (PDI), and zeta potential of Fe‐SH@Fn. Furthermore, the morphology of Fe‐SH@Fn was examined using transmission electron microscope (TEM) and scanning electron microscope (SEM). The chemical composition of the Fe‐SH@Fn was analyzed by EDS. Additionally, the structure of Fe‐SH@Fn was characterized using UV‐vis, FTIR and XPS.

### 4.5 In Vitro SH Release From Fe‐SH@Fn

The GSH‐responsive release behavior of Fe‐SH@Fn was evaluated in vitro using a dialysis method under varying concentrations of GSH (0‐10 mM). At predetermined time intervals (1–24 h), aliquots of the release medium were collected and replaced with fresh medium. The amount of SH released was quantified by measuring the absorbance at 515 nm using a spectrophotometer, with reference to a standard calibration curve.

### 4.6 In Vitro Fenton Performance of Fe‐SH@Fn

The degradation of MB was used to evaluate the Fenton reaction activity. Specifically, a mixture containing MB, H_2_O_2_, and Fe‐SH@Fn, with or without GSH, was reacted under specified conditions. After reaction, the supernatant was collected and the absorbance at 660 nm was measured to quantify MB degradation. Furthermore, ESR spectroscopy was used to confirm the generation of •OH.

### 4.7 Cell Culture

The HL‐60 and C1498 cells were purchased from the Xiamen Immocell Biotechnology Co., Ltd. HL‐60 cells were cultured in IMDM, while C1498 cells were cultured in DMEM. Both media were supplemented with FBS (10% v/v), penicillin (100 units/mL), and streptomycin (100 units/mL). All cells were cultured in an incubator with 37°C in a humidified 5% CO_2_. In addition, before cell administration, all drugs are filtered through a 0.22 µm filter membrane to remove bacteria.

### 4.8 Cell Targeting Ability of Fe‐SH@Fn

To track the cellular internalization, Fe‐SH@Fn nanoparticles were labeled with FITC. HL‐60 and C1498 cells at a density of 2 × 10^5^/well were seeded in 24‐well plates, while HUVEC cells at a density of 5 × 10^4^/well were seeded in 24‐well plates. After 24 h of culture, cells were incubated with Fe‐SH@Fn‐FITC for 2 h, and evaluated using fluorescence microscopy. In a parallel experiment, cells were pretreated with unlabeled Fn for 1 h to block CD71 before adding Fe‐SH@Fn‐FITC, followed by the same imaging procedure.

### 4.9 In Vitro Cytotoxicity of Fe‐SH@Fn

Briefly, HL‐60 and C1498 cells were seeded into 96‐well plates at a density of 3 × 10^4^ cells per well and treated with various formulations. After 24 h of incubation, the cells were incubated with CCK‐8 reagent for 1 h. The optical density (OD) at 450 nm was measured using a microplate reader.

### 4.10 Intracellular ROS, GSH, Fe^2+^, and MDA Detection

HL‐60 cells were seeded in a 24‐well plate and cultured overnight. Subsequently, the cells were treated with different formulations (Fe^3+^: 2 µg/mL and SH: 0.4 µg/mL). After 24 h, the cells were harvested and centrifuged at 1200 rpm for 3 min. Intracellular ROS levels were measured using a DCFH‐DA assay kit. Similarly, intracellular levels of GSH, Fe^2^^+^, and MDA were determined using designative assay kits.

### 4.11 Intracellular LPO Level Assay

HL‐60 cells were seeded and incubated in 24‐well plate for 24 h, and then incubated with different formulations. After 24 h, the cells were stained with LPO probe C11‐BODIPY581/591 for 30 min and washed with PBS for three times. Finally, the fluorescent images were achieved by fluorescence microscope.

### 4.12 Detection of Mitochondrial Function

Following treatment with the respective formulations, HL‐60 cells were collected and stained using the JC‐1 mitochondrial membrane potential assay kit. The cells were then examined and imaged under a fluorescence microscope, and further analyzed by flow cytometry. In addition, changes in mitochondrial morphology were observed using Bio‐TEM.

### 4.13 Western Blot Assay

Briefly, the treated HL‐60 cells were collected and lysed in RIPA lysis buffer for total protein extraction. Subsequently, the harvested proteins were quantified using BCA protein assay kit, separated on 10% SDS‐PAGE, and transferred onto PVDF membrane. Then, the PVDF membrane were blocked with 5% BSA and incubated with the primary antibodies overnight at 4°C. After that, the PVDF membrane were washed with TBST and incubated with goat anti‐rabbit IgG H&L (1:2000) for 1.5 h at room temperature. Finally, the bands were visualized with an enhanced chemiluminescence detection system, and then quantified by using ImageJ software.

### 4.14 CRT, HMGB1, and ATP Detection

Firstly, collect the processed cells and fixed with 4% paraformaldehyde, blocked with 10% BSA, and then incubated with anti‐CRT and anti‐HMGB1 antibody, followed by stained with Cy3‐conjugated secondary antibody, and DAPI respectively, and the level of CRT and HMGB1 was measured by flow cytometry and confocal microscope assay. Moreover, the extracellular release of HMGB1 quantified via ELISA kits, and the secretion of ATP was detected by enhanced ATP assay kit.

### 4.15 Tumor Targeting Effect of Fe‐SH@Fn

All animal procedures were conducted in strict accordance with the Guide for the Care and Use of Laboratory Animals(National Research Council, 2011) and approved by the Sichuan Provincial People's Hospital Medical Ethics Committee (SPPHMEC, Approval No. 2024–092). Fe‐ICG@Fn was synthesized by substituting SH with ICG. Then, Fe‐ICG@Fn were injected intravenously into the C1498 tumor‐bearing mice. Subsequently, the In vivo fluorescence images were captured through the In vivo imaging system. For ex vivo fluorescence imaging, the tumor‐bearing mice were intravenously injected Fe‐ICG@Fn. After 6 h, the mice were sacrificed, the tumor, heart, liver, spleen, lung, and kidney were excised and imaged.

### 4.16 In Vivo Therapeutic Effect of Fe‐SH@Fn

AML transplant models were established by subcutaneous injection of C1498 cells into C57BL/6 mice. Tumor‐bearing mice were randomly divided into five groups as follows (*n* = 5): (G1) Control; (G2) FeCl_3_; (G3) SH; (G4) Fe‐SH; (G5) Fe‐SH@Fn. When the tumor volume reaches 200 mm^3^, formulations were intravenously administered (SH‐equivalent 2 mg/kg, FeCl_3_‐equivalent 10 mg/kg) on days 1, 3, and 5. Body weight and tumor volume (Volume = length × width^2^ × 0.5) were monitored daily from initial treatment. Mice were sacrificed on day 7 for tumor and organs collection.

AML orthotopic models were established in female C57BL/6 mice by intravenous injection of C1498 cells. Seven days after inoculation, model establishment was confirmed by peripheral leukocytosis (>5×10^9^/L leukocyte count). Mice were then randomly assigned to treatment cohorts (*n* = 5) and received intravenous treatments (SH‐equivalent 2 mg/kg, FeCl_3_‐equivalent 10 mg/kg) on days 1, 3, and 5. Body weights were monitored until day 7. Blood samples and major organs‐including heart, liver, spleen, lung, and kidney‐were collected for histopathological analysis in day 14.

### 4.17 H&E and TUNEL Staining

The tumor, heart, liver, spleen, lung and kidney tissues were fixed in 4% paraformaldehyde, embedded in paraffin, and sectioned into 5 µm slices. Sections were subjected to H&E and TUNEL staining according to standard protocols. For nuclear visualization, tissue slices were counterstained with DAPI for 10 min. Stained slides were imaged using a confocal laser scanning microscope (CLSM).

### 4.18 Immunohistochemical and Immunofluorescence Staining

Tumor sections were incubated with primary antibodies including anti‐Ki67, anti‐SLC7A11, anti‐FTH1, anti‐GPX4, anti‐CRT for 12 h, followed by incubation with Alexa Fluor 488/Cy3‐conjugated secondary antibodies for 1 h. Cell nuclei were counterstained with DAPI prior to CLSM imaging.

### 4.19 In Vivo Immune Activation Effect Evaluation

Lymph nodes, tumors, and spleens were harvested from treated mice and digested in collagenase IV (0.3 mg/mL) at 37°C for 1 h to obtain single‐cell suspensions. Cells were blocked with CD16/CD32 antibody for 15 min, and then stained with Fixable Viability Stain 620 at 4°C for 15 min. For flow cytometric analysis, T cells were labeled with anti‐CD45, CD3, CD4, and CD8 antibodies; regulatory T cells (Tregs) were stained with anti‐CD45, CD3, CD4, and Foxp3 antibodies; mature dendritic cells (DCs) were labeled with anti‐CD45, MHCII, CD11c, CD80, and CD86 antibodies. All samples were analyzed by flow cytometry.

### 4.20 Safety Assay

Blood and serum samples were collected from tumor‐bearing mice to evaluate hematological parameters, including white blood cells (WBC), red blood cells (RBC), lymphocytes (Lymph), granulocytes (Gran), and platelets (PLT). Serum was further analyzed for liver function markers, aspartate aminotransferase (AST) and alanine aminotransferase (ALT), as well as renal function indicators, urea and creatinine (Crea).

### 4.21 Statistical Analysis

All data were performed, including one‐way ANOVA, and unpaired t‐test, using GraphPad Prism 10, ImageJ, and Origin 2021 software. Before statistical analysis, data were examined for outliers and tested for normality and homogeneity of variance. No data transformation or normalization was applied unless otherwise specified. Data are presented as mean ± standard deviation (mean ± SD). The sample size (n) for each experiment is indicated in the corresponding figure legends. Differences were considered statistically significant at *p* < 0.05. Statistical significance is denoted as follows: ns, not significant (*p* ≥ 0.05), ^*^ *p* < 0.05, ^**^ *p* < 0.01, and ^***^ *p* < 0.001.

## Conflicts of Interest

The authors declare no conflicts of interest.

## Supporting information




**Supporting File**: advs74704‐sup‐0001‐SuppMat.docx.

## Data Availability

The data that support the findings of this study are available on request from the corresponding author. The data are not publicly available due to privacy or ethical restrictions.
