# Supplementary material for: AML‐Targeted Metal‐Polyphenol Nanoplatform Induces Ferroptosis‐ICD Cascade for Antitumor Immunity Boosting
Source: Adv Sci (Weinh). 2026 Mar 3;13(27):e20544. doi: 10.1002/advs.202520544 (PMC13170192; doi:10.1002/advs.202520544)
Supplement: Supplementary file 1 — Supporting File: advs74704‐sup‐0001‐SuppMat.docx. [file ADVS-13-e20544-s001.docx]

Supporting Information

**AML-Targeted Metal-Polyphenol Nanoplatform Induces Ferroptosis-ICD Cascade for Antitumor Immunity Boosting**

Shangqin Yang^a,c^, Jingxuan Wang^b^, Kerong Tu^b^, Xiaobing Huang^a^, Liangliang Lv^c^, Mingjie Peng^d^, Qiqi Xu^b^, Hongmei Liu^b^, Qiang Sun^a,b,*^, Lulu Cai^b,*^

*^a^Department of Hematology, Sichuan Provincial People’s Hospital, School of Medicine, University of Electronic Science and Technology of China, Chengdu 610072, China.*

*^b^Department of Pharmacy, Personalized Drug Research and Therapy Key Laboratory of Sichuan Province, Sichuan Provincial People’s Hospital, University of Electronic Science and Technology of China, Chengdu 610072, China.*

*^c^School of Pharmacy, Southwest Medical University, Luzhou 646000, China.*

*^d^College of Pharmacy, Chengdu University, Chengdu 610106, China.*

***Corresponding Authors.**

E-mail addresses: [cailulu@med.uestc.edu.cn](mailto:cailulu@med.uestc.edu.cn) (Lulu Cai), sunqiang@uestc.edu.cn (Qiang Sun).

**1. Supplementary Figures**

**
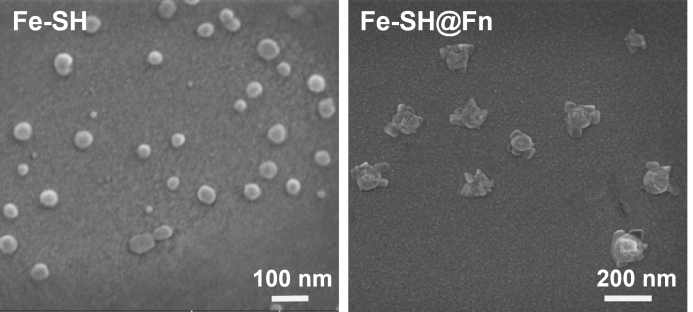
**

**Figure S1.** SEM images of Fe-SH and Fe-SH@Fn.


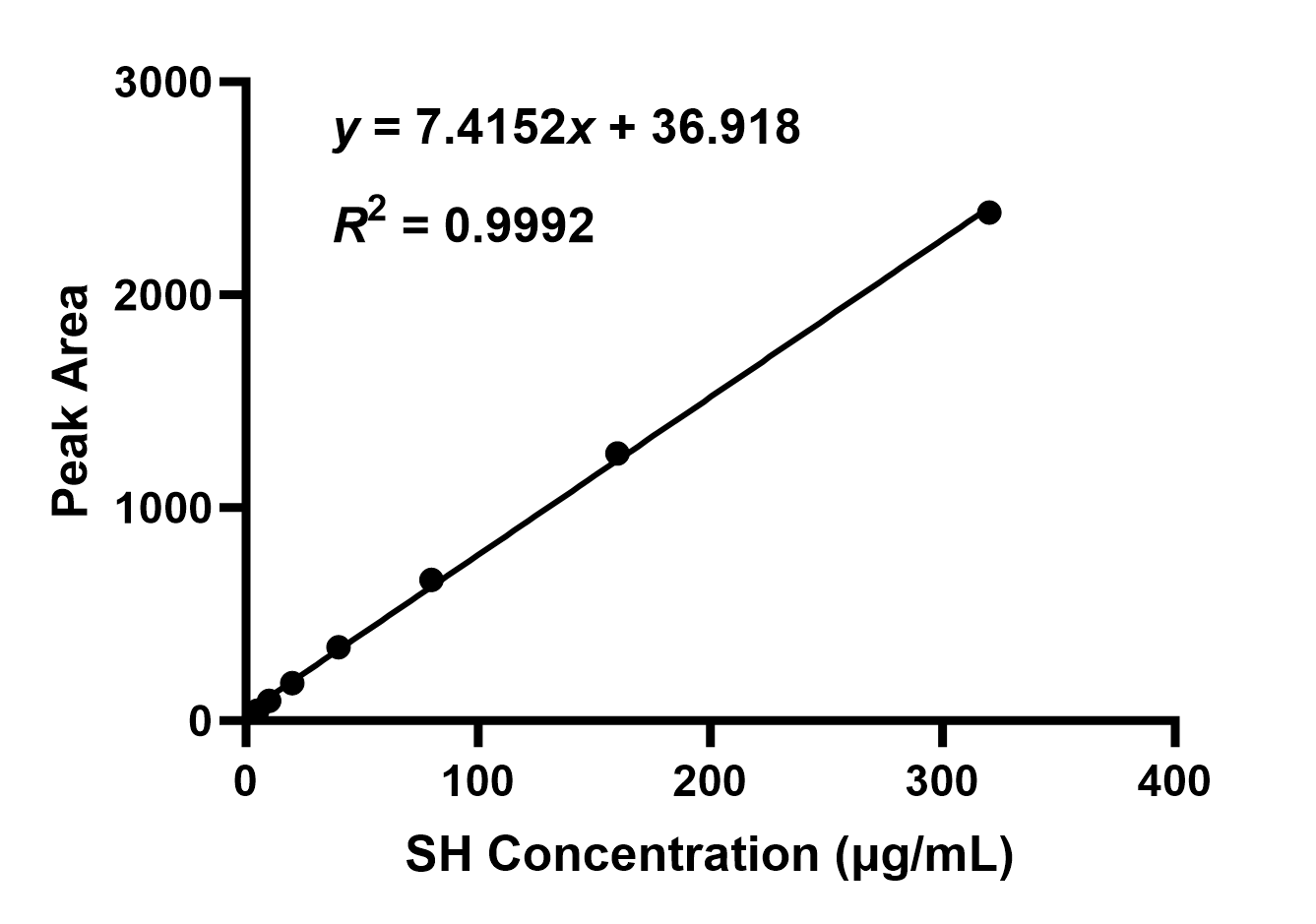


**Figure S2.** The standard curve of SH by HPLC (*y* = 7.4152*x* + 36.918, *R*^2^ = 0.9992).


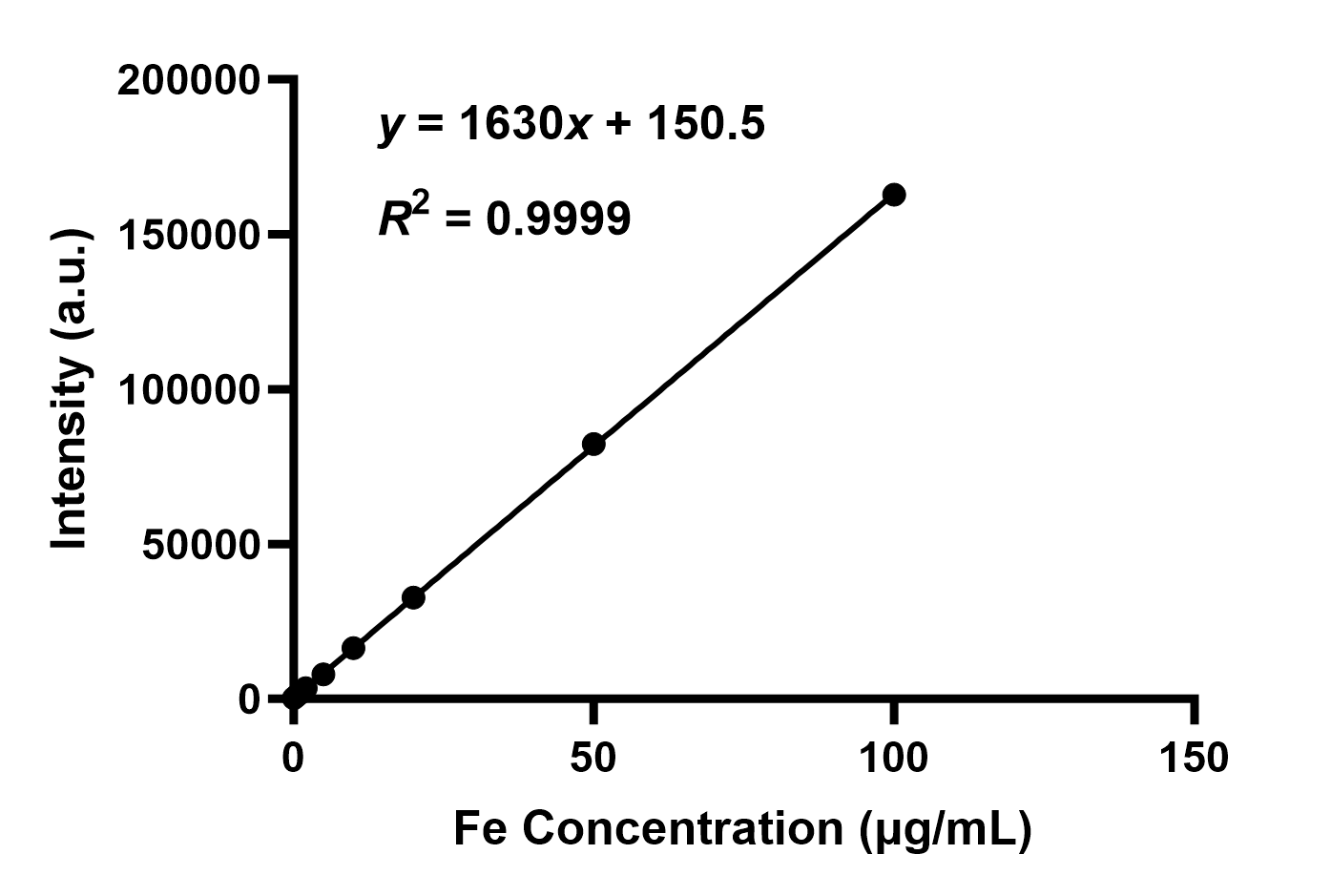


**Figure S3.** The standard curve of Fe by ICP-OES (*y* = 1630*x* + 150.5, *R*^2^ = 0.9999).


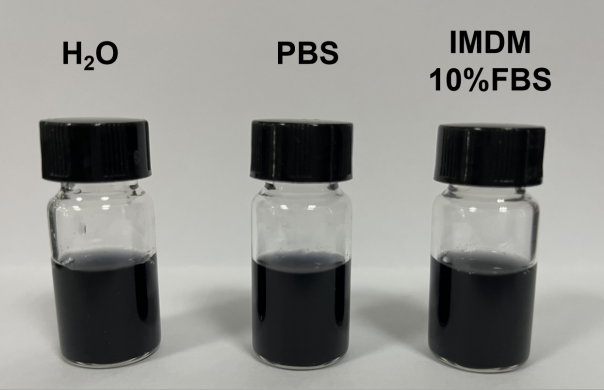


**Figure S4.** Appearance of Fe-SH@Fn in water, PBS, and IMDM medium (10% FBS), respectively one week after preparation.


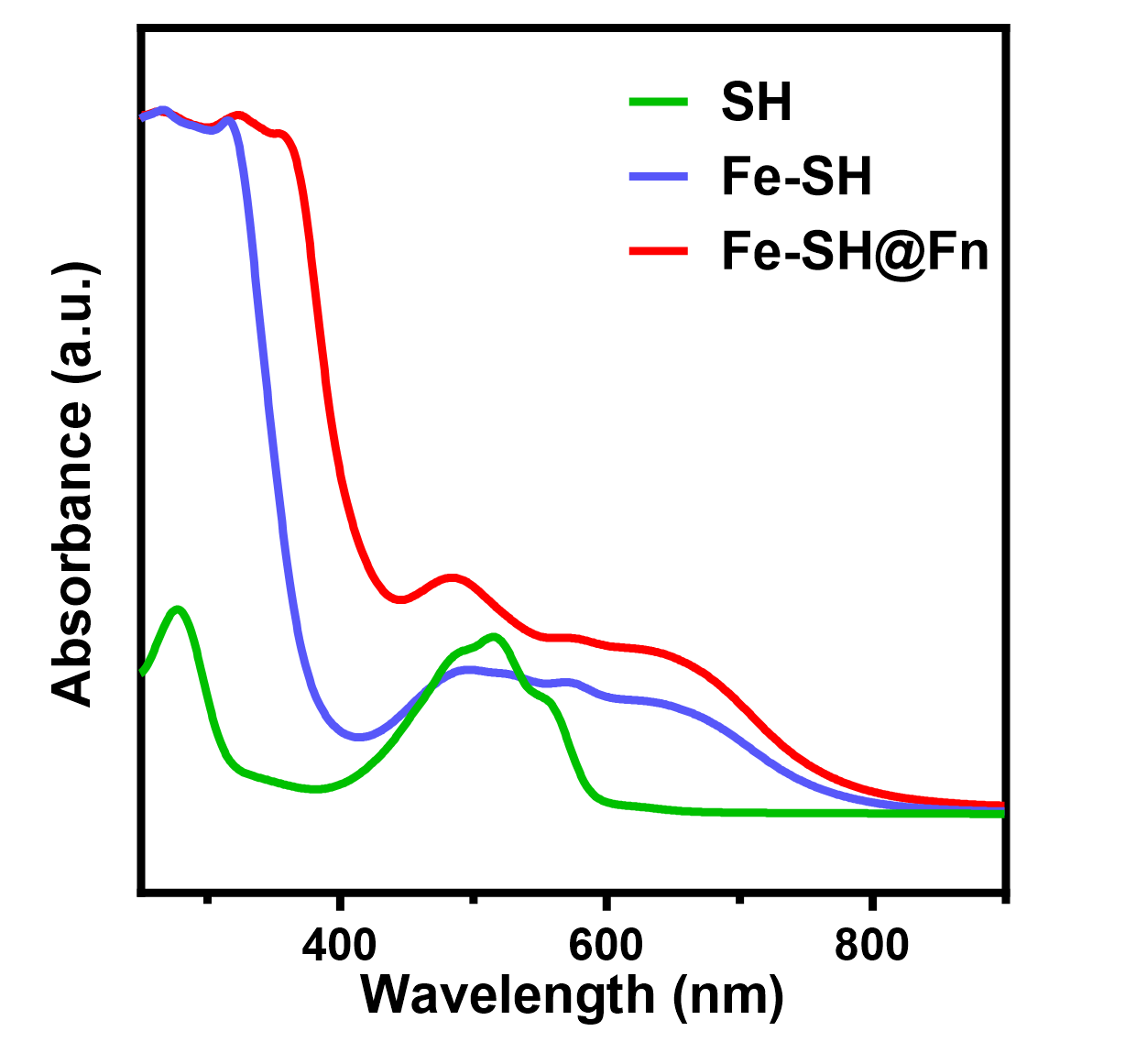


**Figure S5.** UV-vis absorption spectra of SH, Fe-SH, and Fe-SH@Fn.


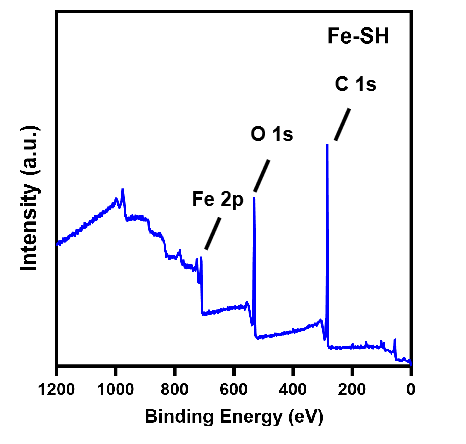


**Figure S6.** XPS full spectrum of Fe-SH.


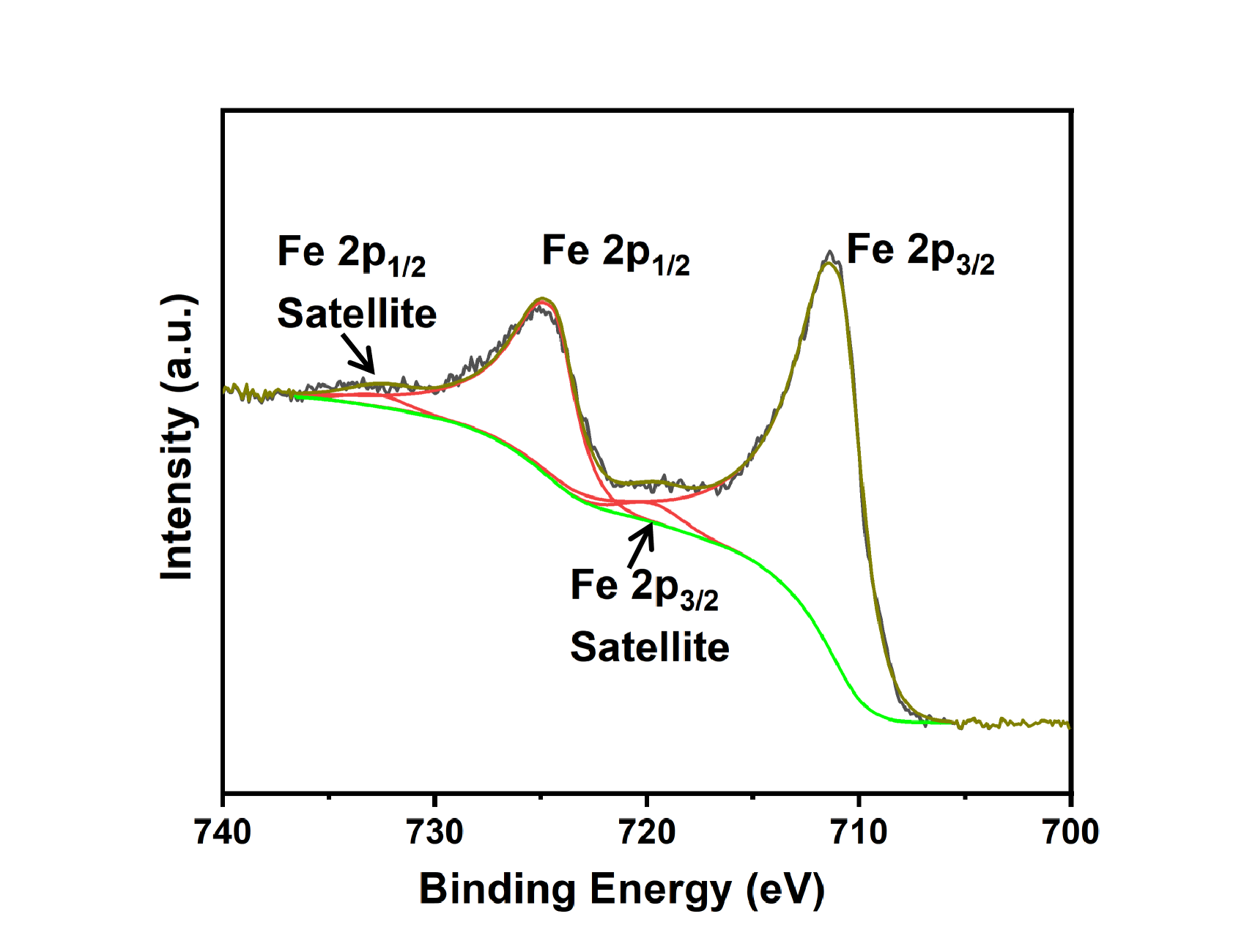


**Figure S7.** High-resolution XPS scans of Fe 2p in Fe-SH@Fn.


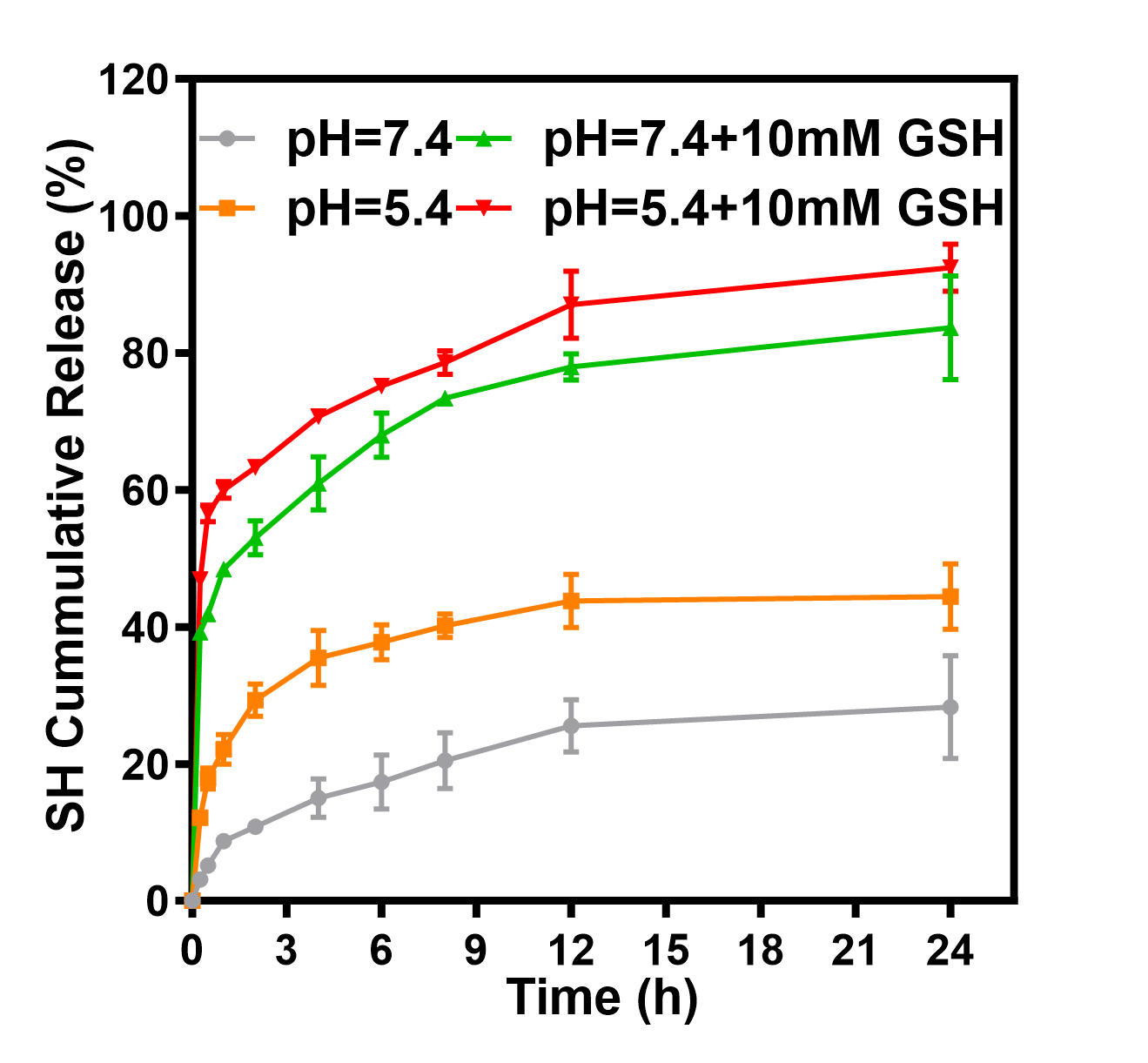


**Figure S8.** Accumulative release of SH from Fe-SH@Fn with and without GSH conditions. Data are presented as mean ± SD (n = 3).


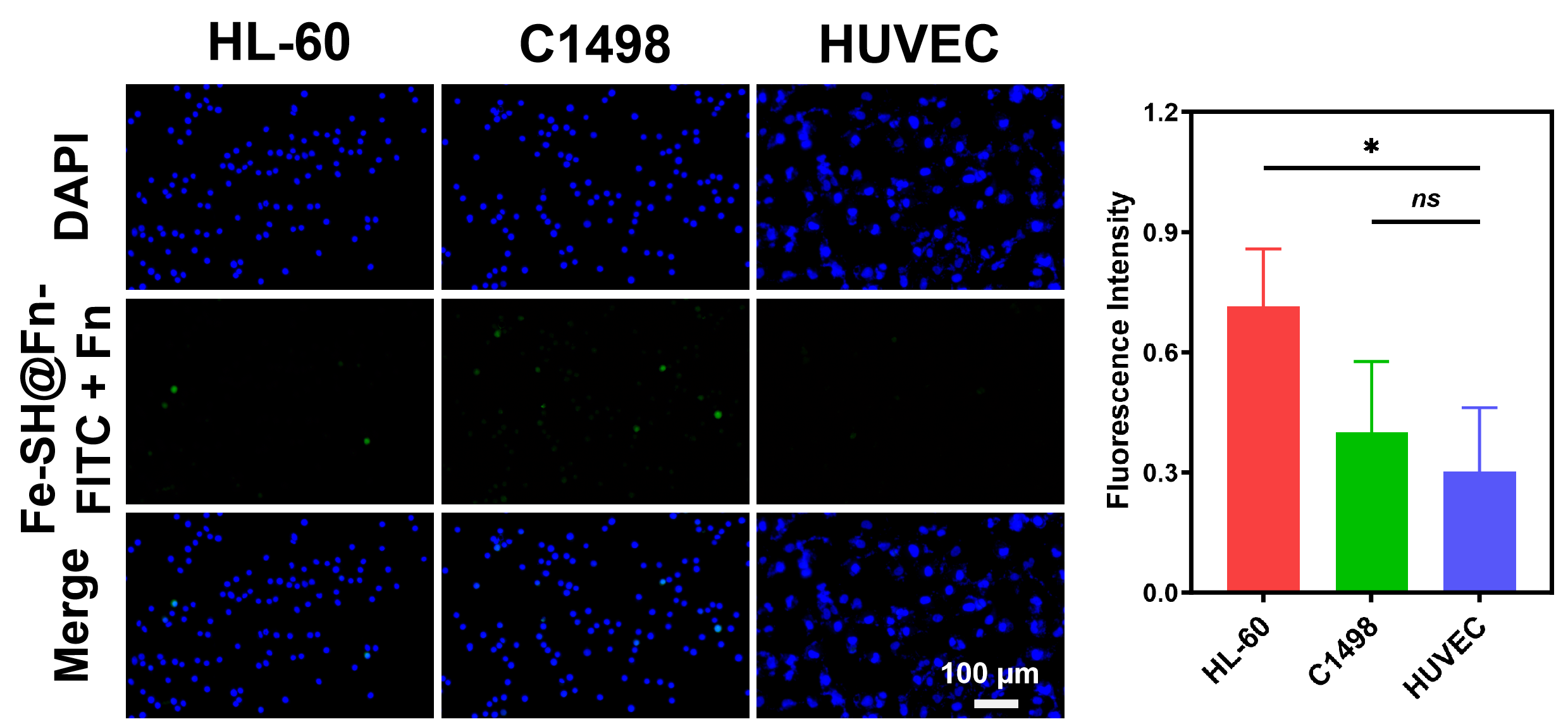


**Figure S9.** The fluorescent images of Fe-SH@Fn-FITC uptake by HL-60, C1498, and HUVEC cells after blocking CD71. Data are presented as mean ± SD (n = 3). Statistical significance was assessed using one-way ANOVA. **p* < 0.05. ns, not significant.


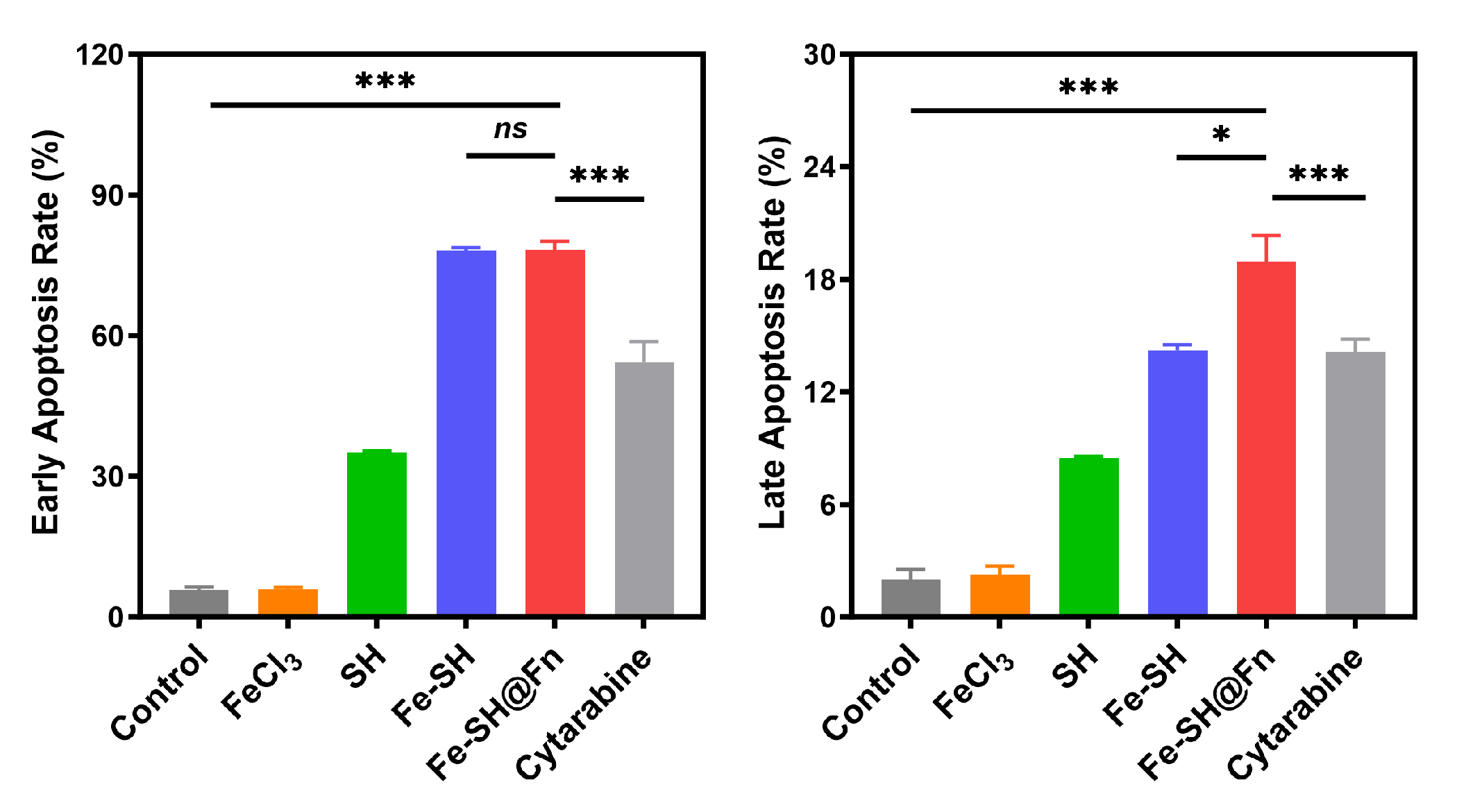


**Figure S10.** Flow cytometry quantitative analysis of early and late apoptosis analysis of HL-60 cells after different treatments. Data are presented as mean ± SD (n = 3). Statistical significance was assessed using one-way ANOVA. ***p* < 0.05, ***p* < 0.01, and ****p* < 0.001. ns, not significant.


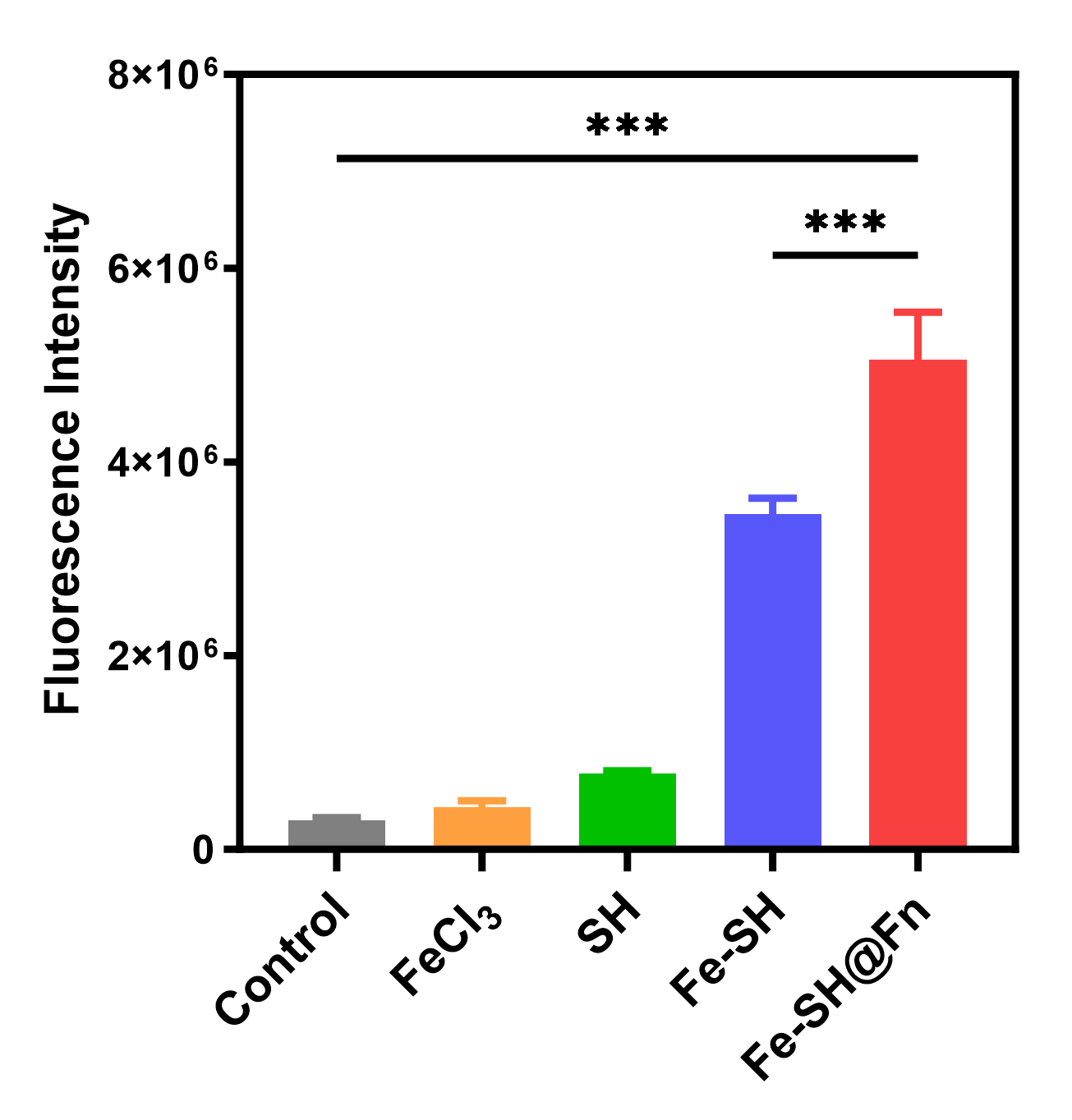


**Figure S11.** Fluorescence quantification of ROS generation in Figure 3A. Data are presented as mean ± SD (n = 3). Statistical significance was assessed using one-way ANOVA. ****p* < 0.001.


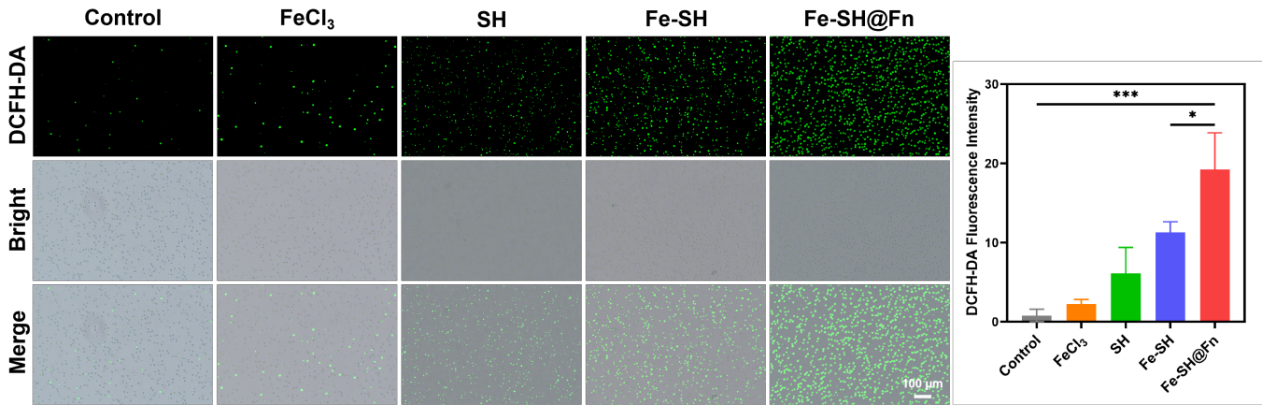


**Figure S12.** ROS production in HL-60 cells and related fluorescence image after different treatments. Data are presented as mean ± SD (n = 3). Statistical significance was assessed using one-way ANOVA. **p* < 0.05, and ****p* < 0.001.


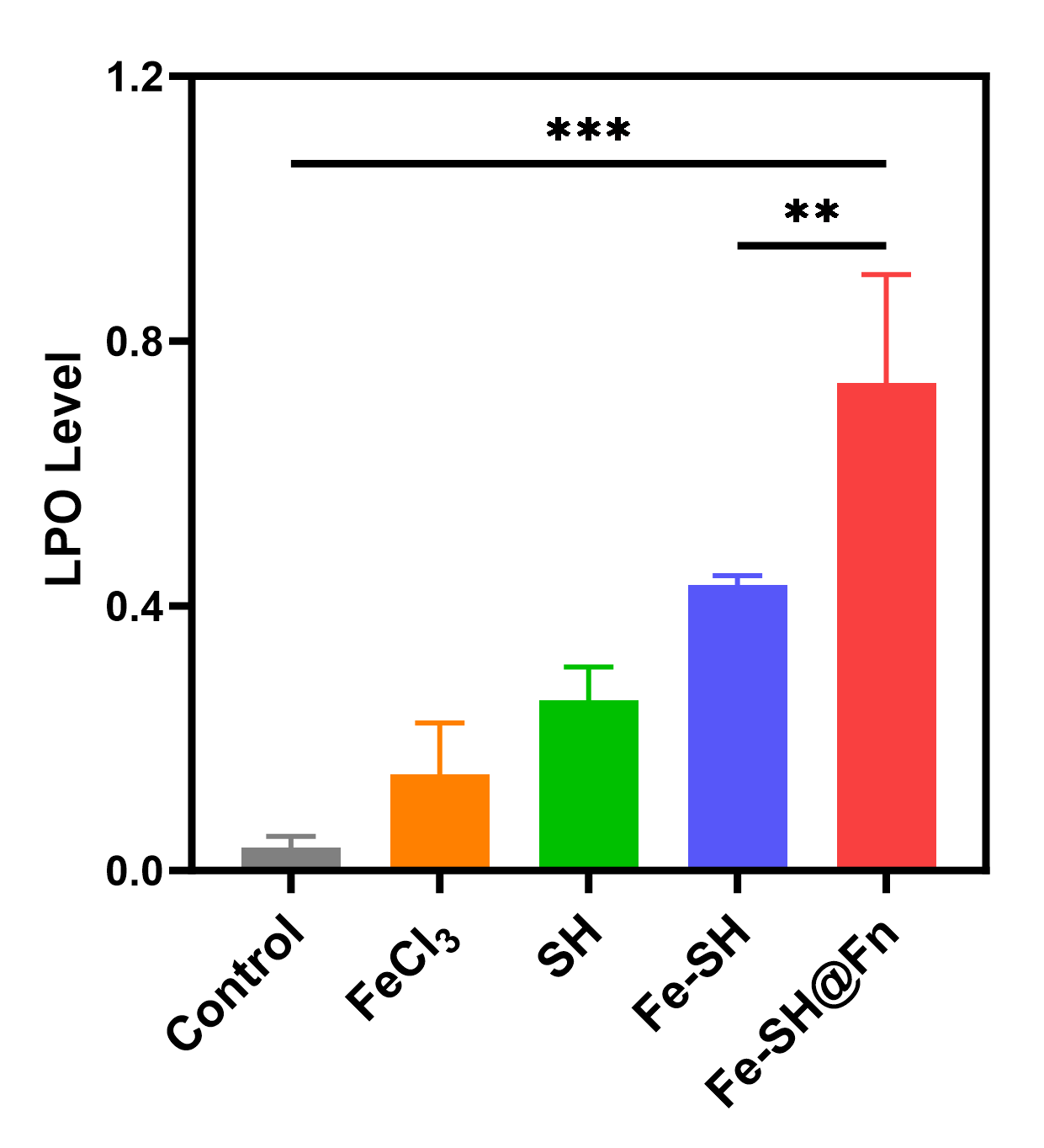


**Figure S13.** Quantitative fluorescence analysis of LPO. Data are presented as mean ± SD (n = 3). Statistical significance was assessed using one-way ANOVA. ***p* < 0.01, and ****p* < 0.001.

**
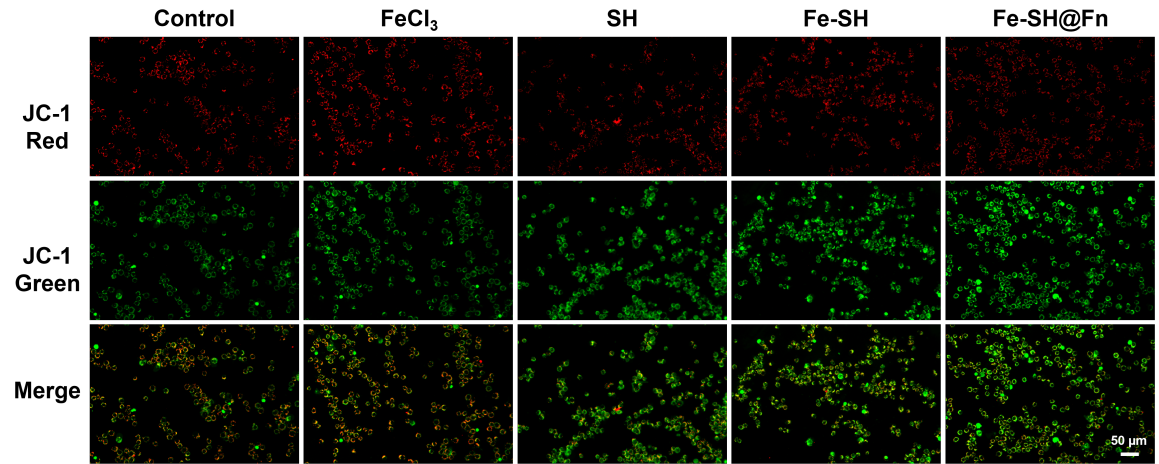
**

**Figure S14.** Fluorescence images of JC-1-stained HL-60 cells after different treatments.

**
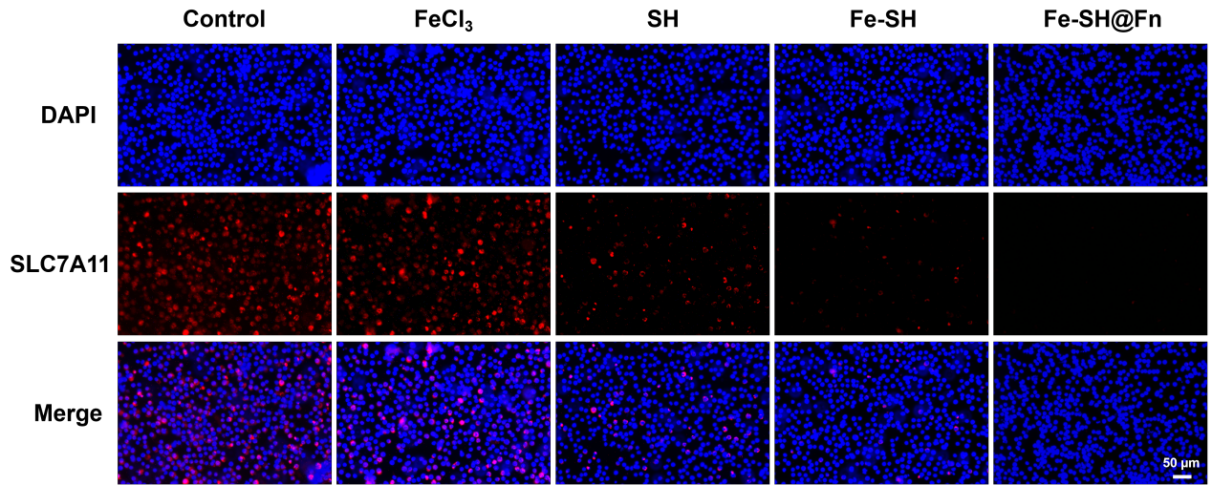
**

**Figure S15.** Immunofluorescence staining images of SLC7A11 in HL-60 cells after different treatments.

**
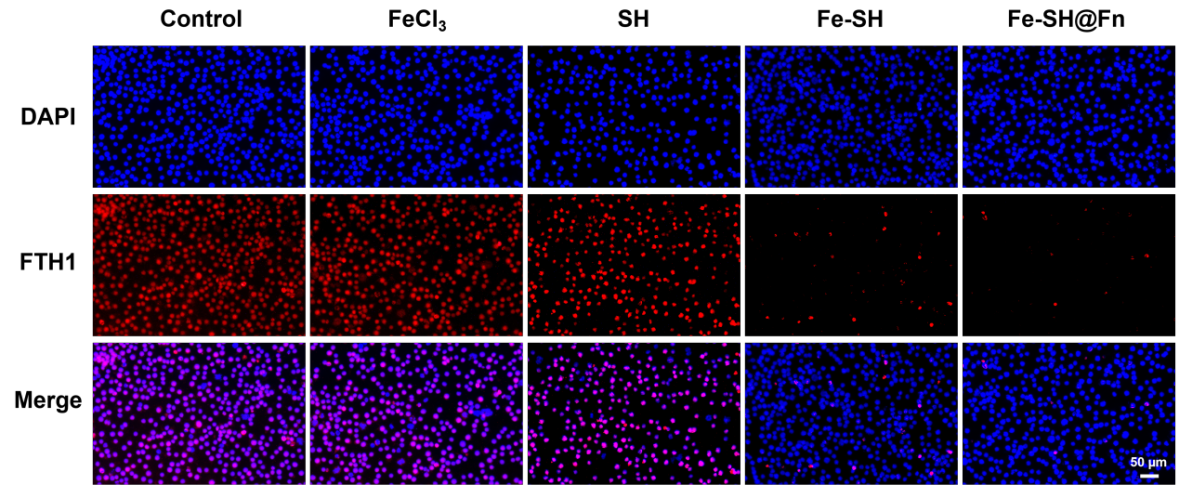
**

**Figure S16.** Immunofluorescence staining images of FTH1 in HL-60 cells after different treatments.


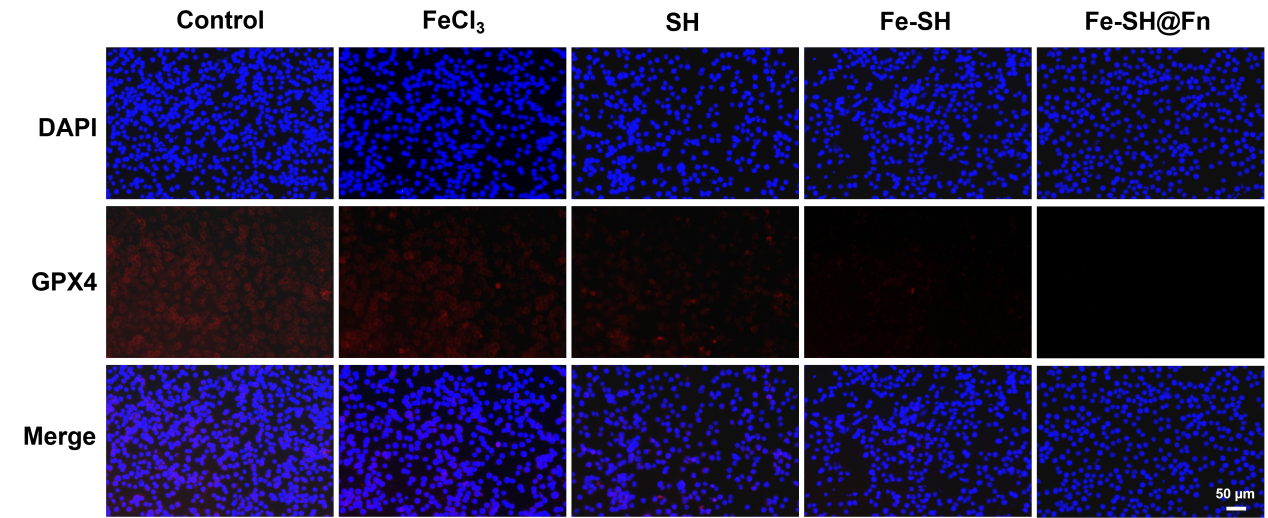


**Figure S17.** Immunofluorescence staining images of GPX4 in HL-60 cells after different treatments.


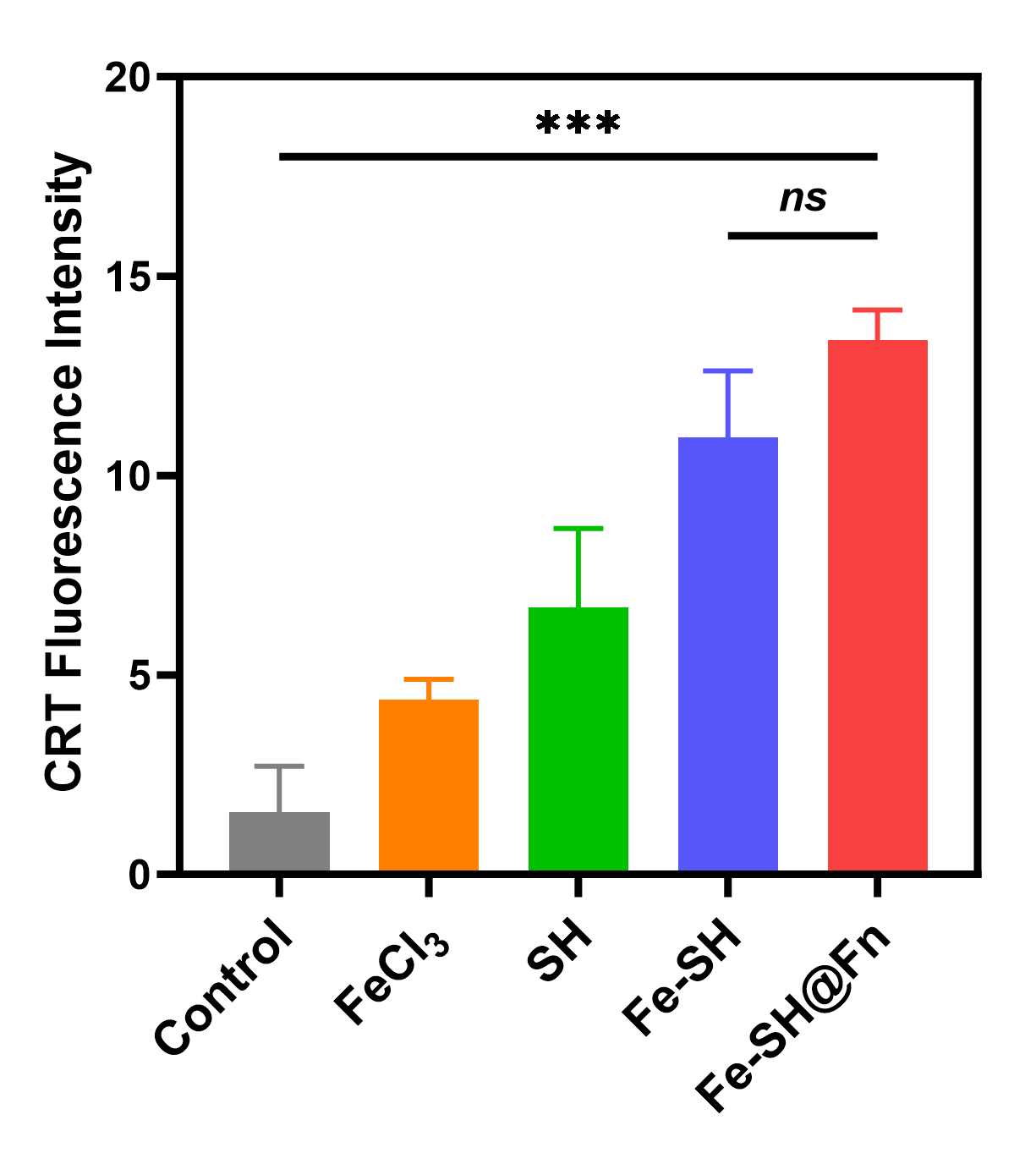


**Figure S18.** Quantitative fluorescence analysis of CRT. Data are presented as mean ± SD (n = 3). Statistical significance was assessed using one-way ANOVA. ****p* < 0.001. ns, not significant.


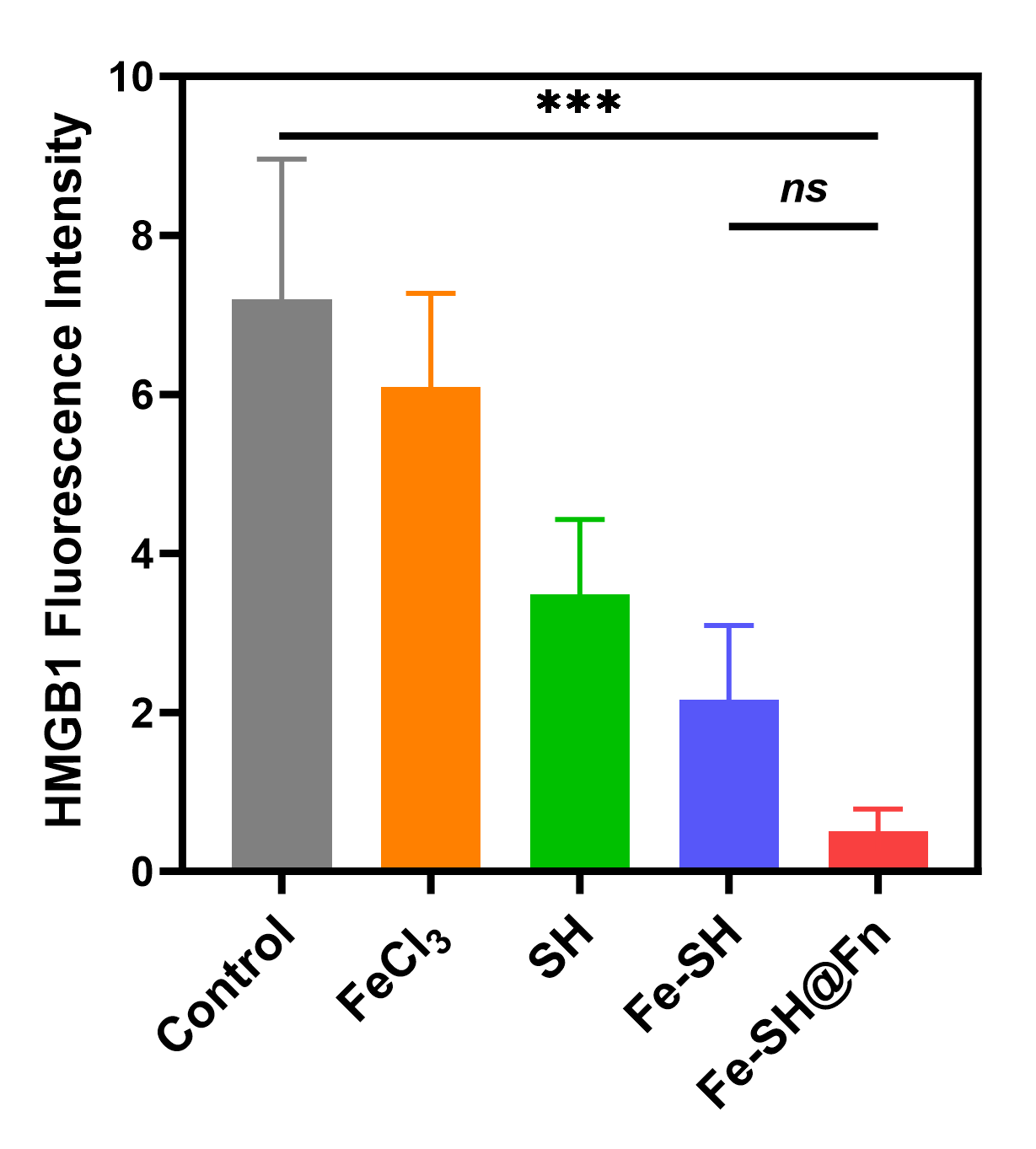


**Figure S19.** Quantitative fluorescence analysis of HMGB1. Data are presented as mean ± SD (n = 3). Statistical significance was assessed using one-way ANOVA. ****p* < 0.001. ns, not significant.


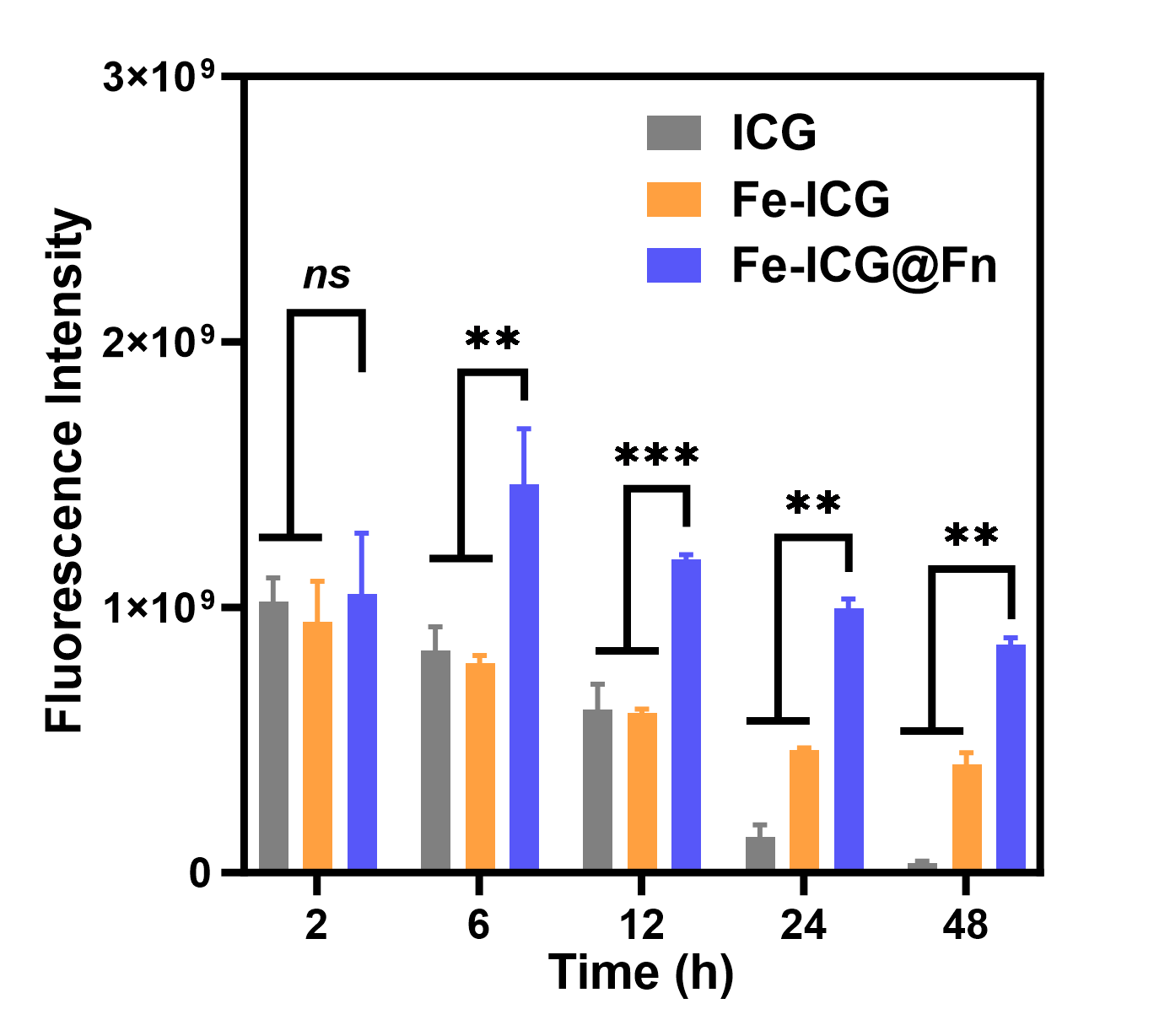


**Figure S20.** Quantitative analysis of fluorescence in the marked tumor region in vivo. Data are presented as mean ± SD (n = 3). Statistical significance was assessed using one-way ANOVA. ***p* < 0.01, and ****p* < 0.001. ns, no significant.


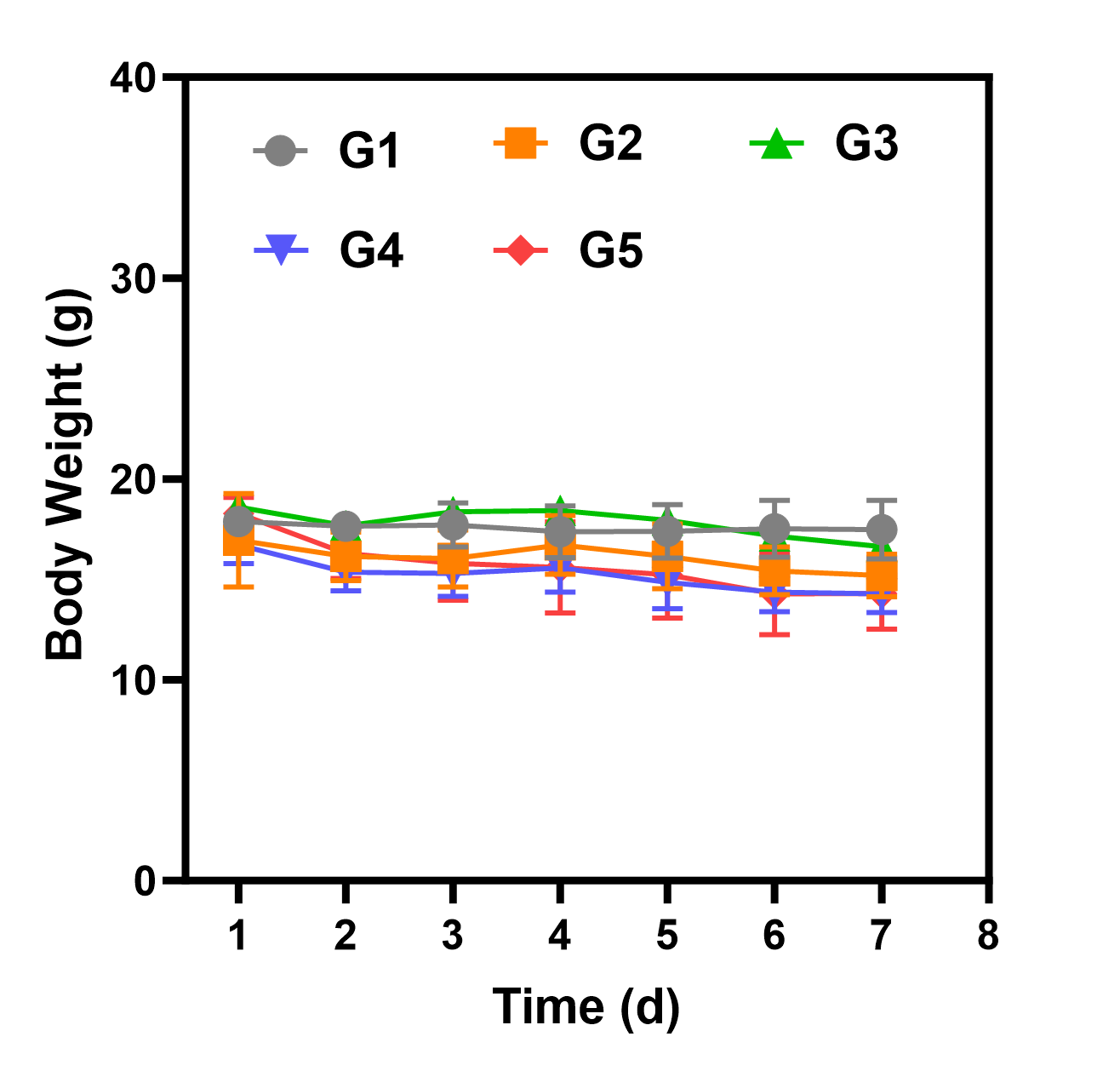


**Figure S21.** Body weight changes of subcutaneous tumor mice over time after different treatments. Data are presented as mean ± SD (n = 5).


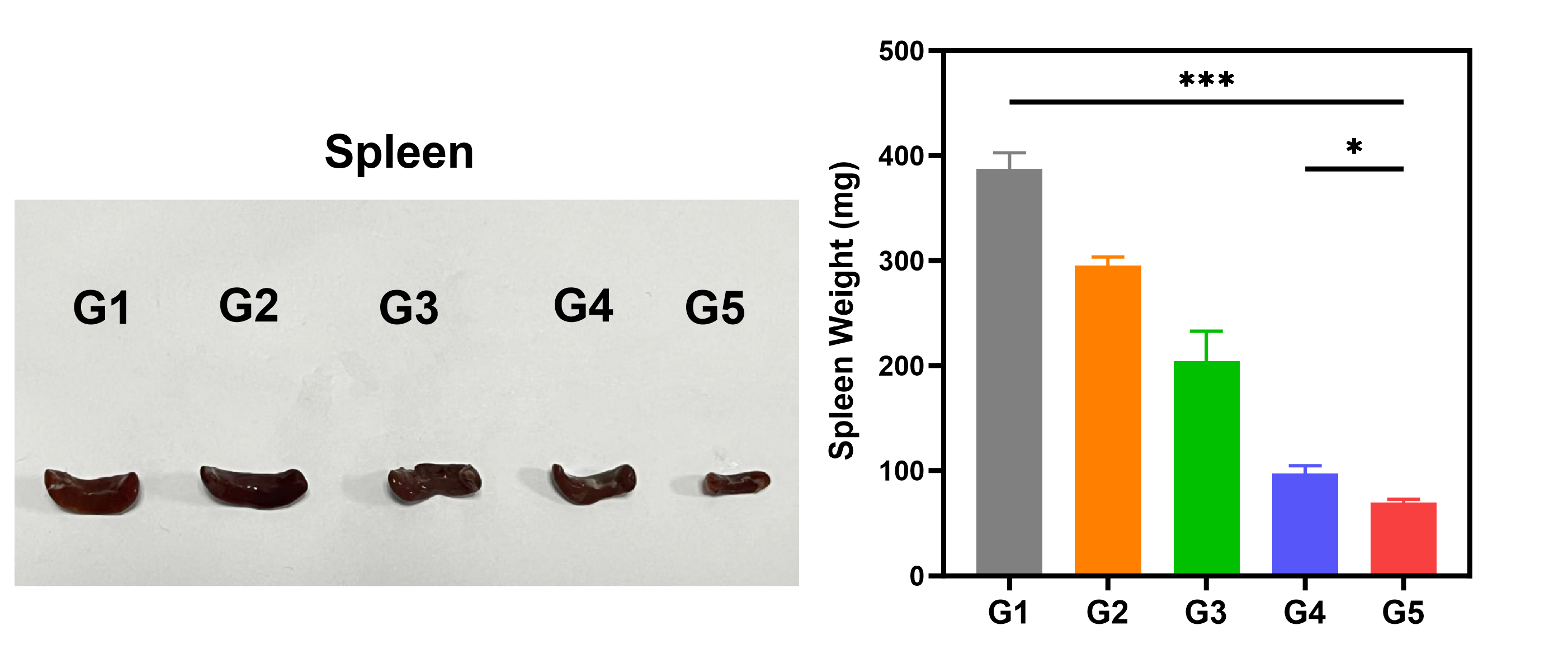


**Figure S22.** Representative images and weight of spleen in subcutaneous tumor mice after different treatments. Data are presented as mean ± SD (n = 3). Statistical significance was assessed using one-way ANOVA. **p* < 0.05, and ****p* < 0.001.


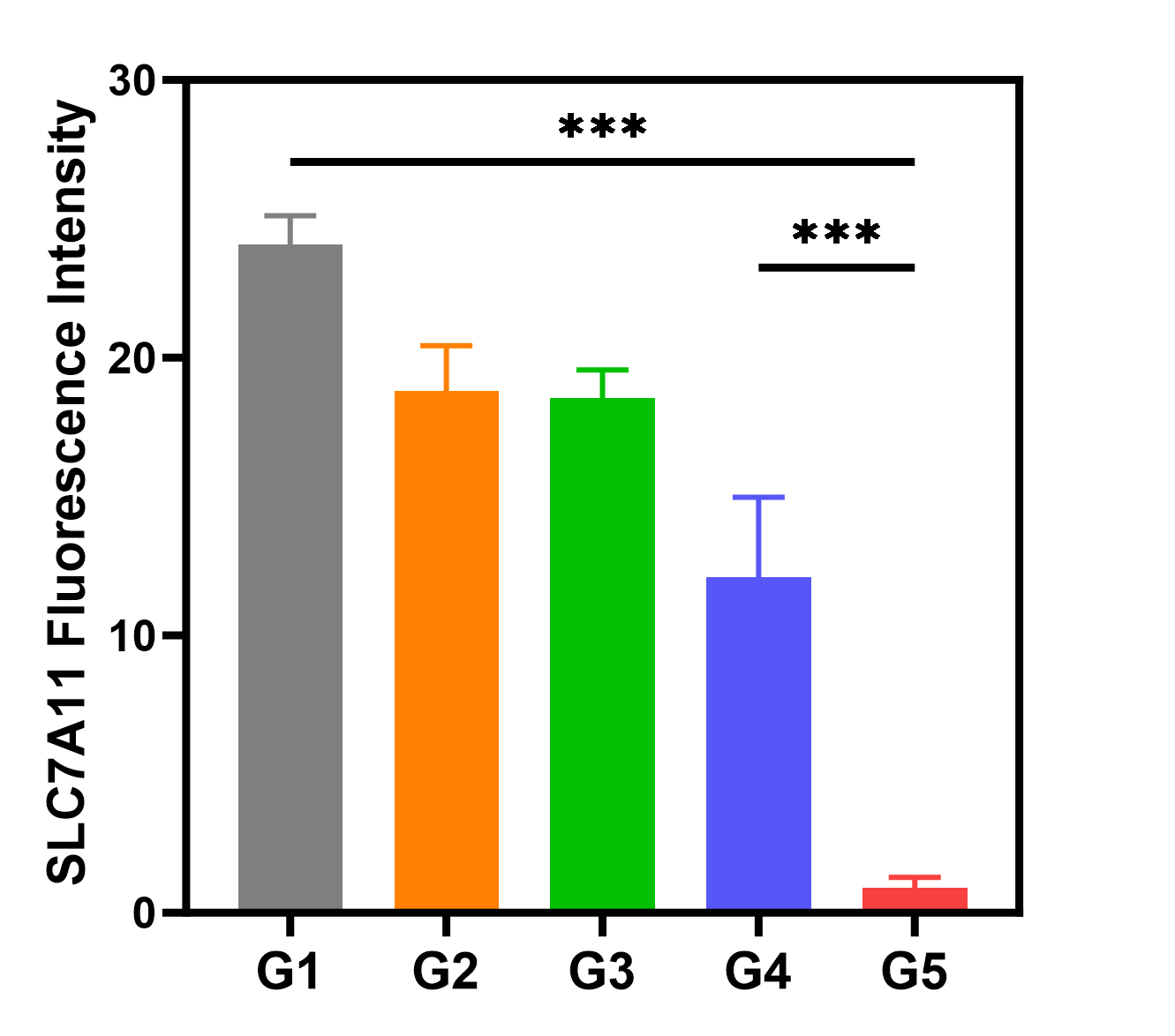


**Figure S23.** Quantitative fluorescence analysis of SLC7A11 in vivo. Data are presented as mean ± SD (n = 3). Statistical significance was assessed using one-way ANOVA. ****p* < 0.001.


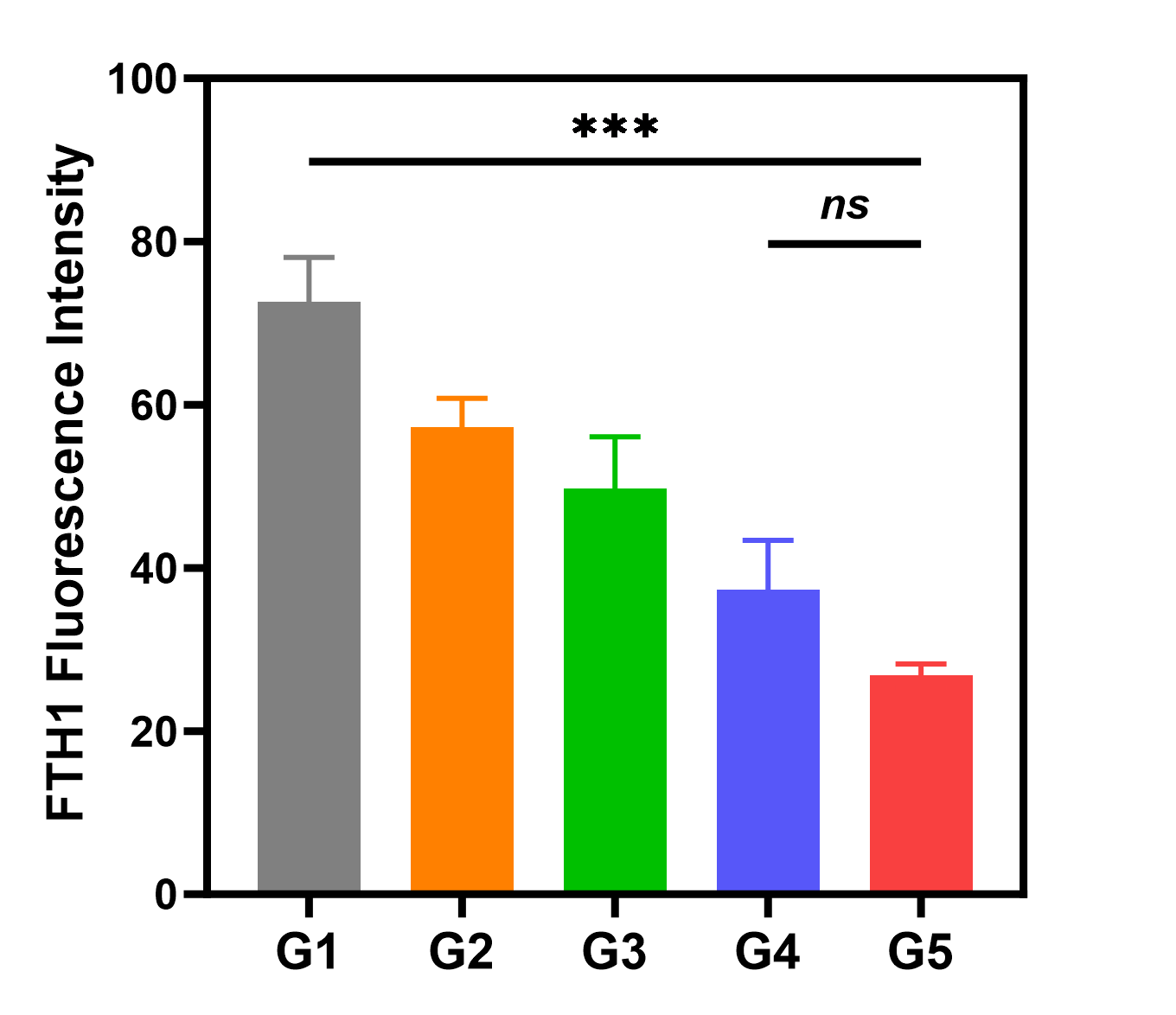


**Figure S24.** Quantitative fluorescence analysis of FTH1 in vivo. Data are presented as mean ± SD (n = 3). Statistical significance was assessed using one-way ANOVA. ****p* < 0.001. ns, not significant.


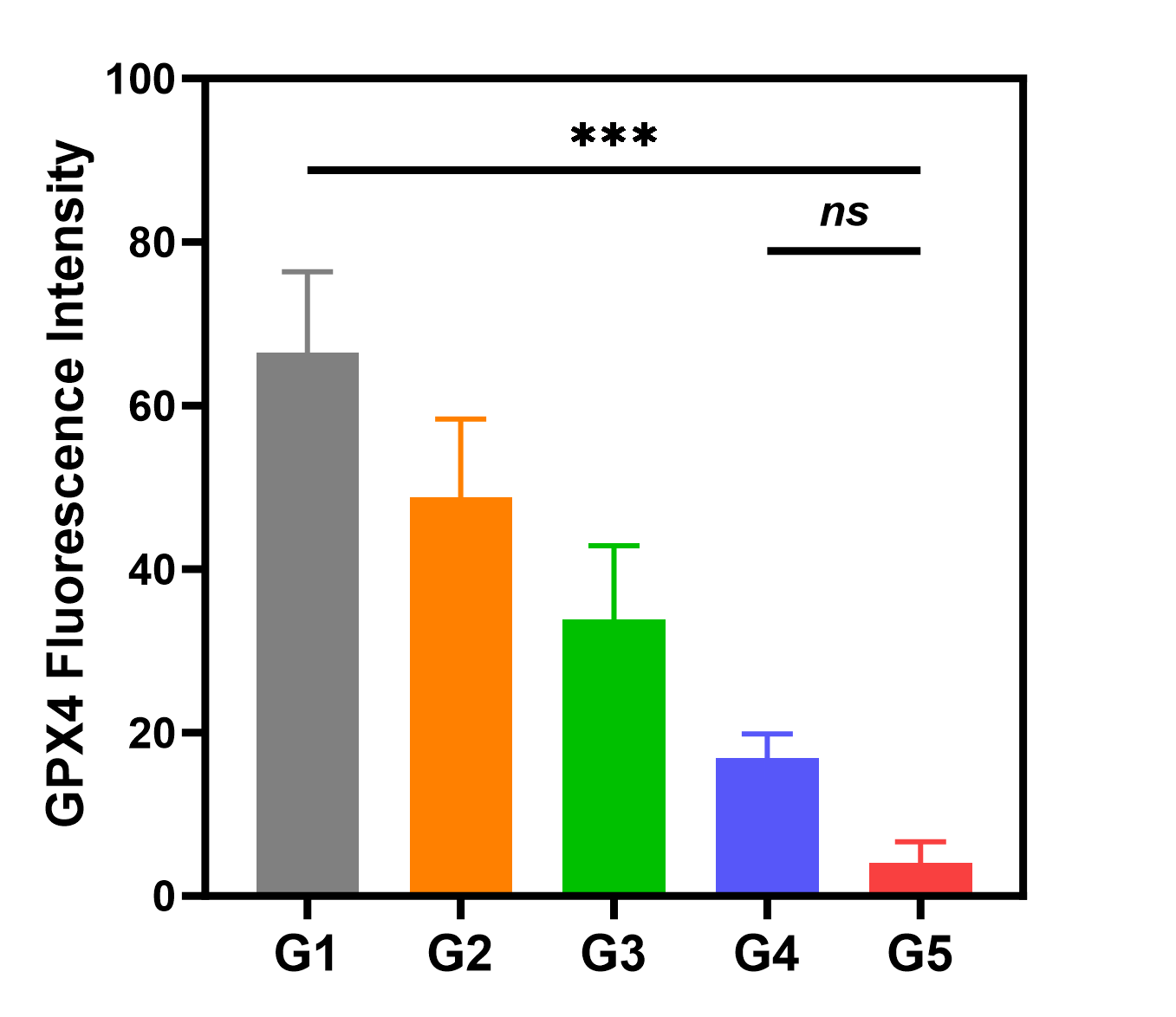


**Figure S25.** Quantitative fluorescence analysis of GPX4 in vivo. Data are presented as mean ± SD (n = 3). Statistical significance was assessed using one-way ANOVA. ****p* < 0.001. ns, not significant.


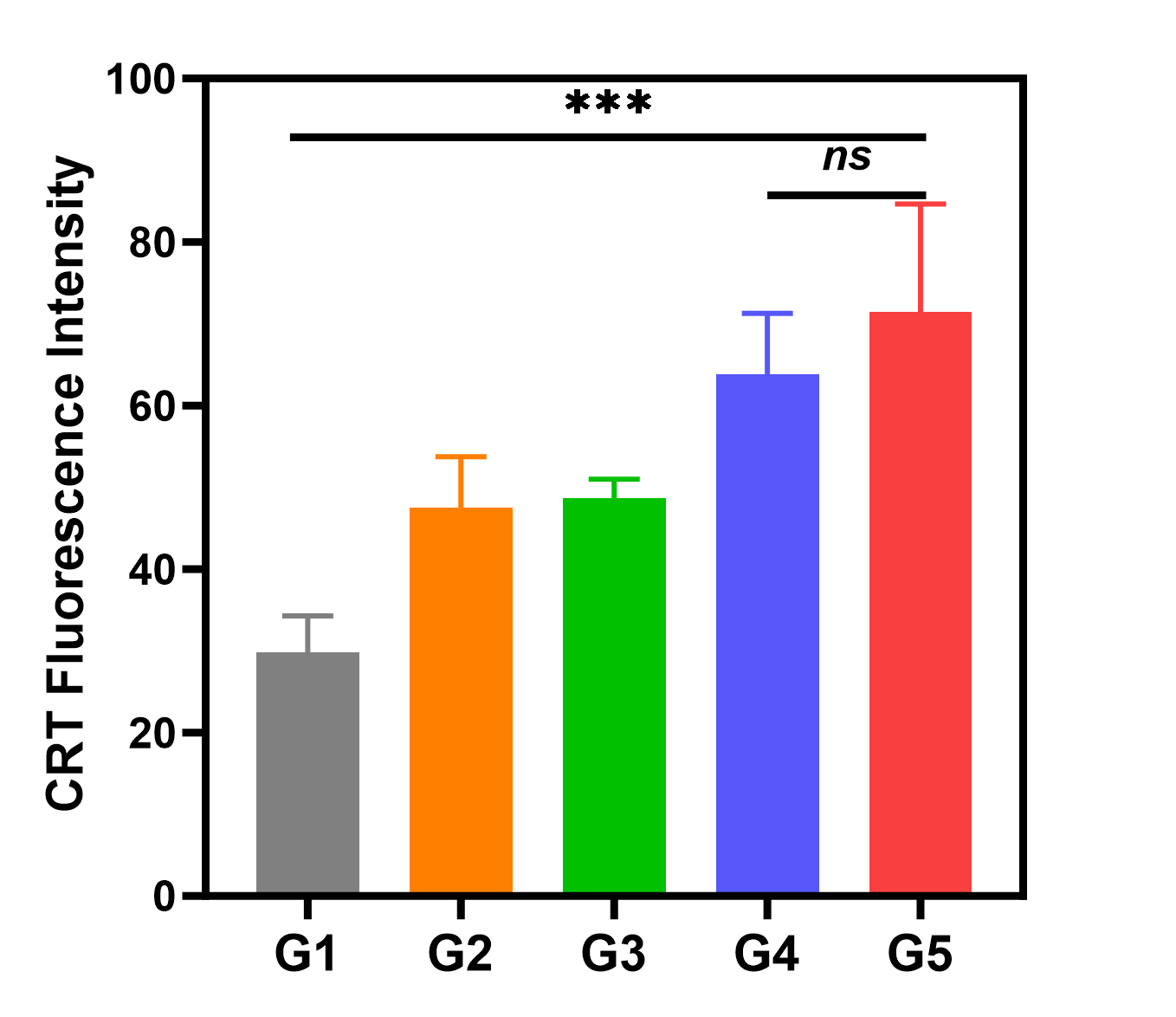


**Figure S26.** Quantitative fluorescence analysis of CRT in vivo. Data are presented as mean ± SD (n = 3). Statistical significance was assessed using one-way ANOVA. ****p* < 0.001. ns, not significant.


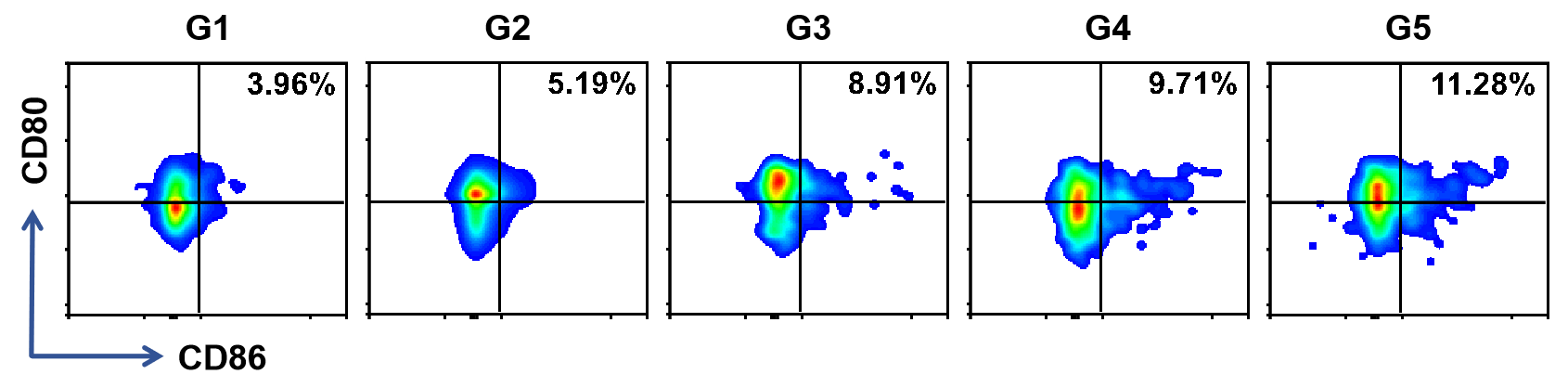


**Figure S27.** Flow cytometry analysis of mature DCs (CD80^+^CD86^+^) in tumor-draining lymph nodes of mice.


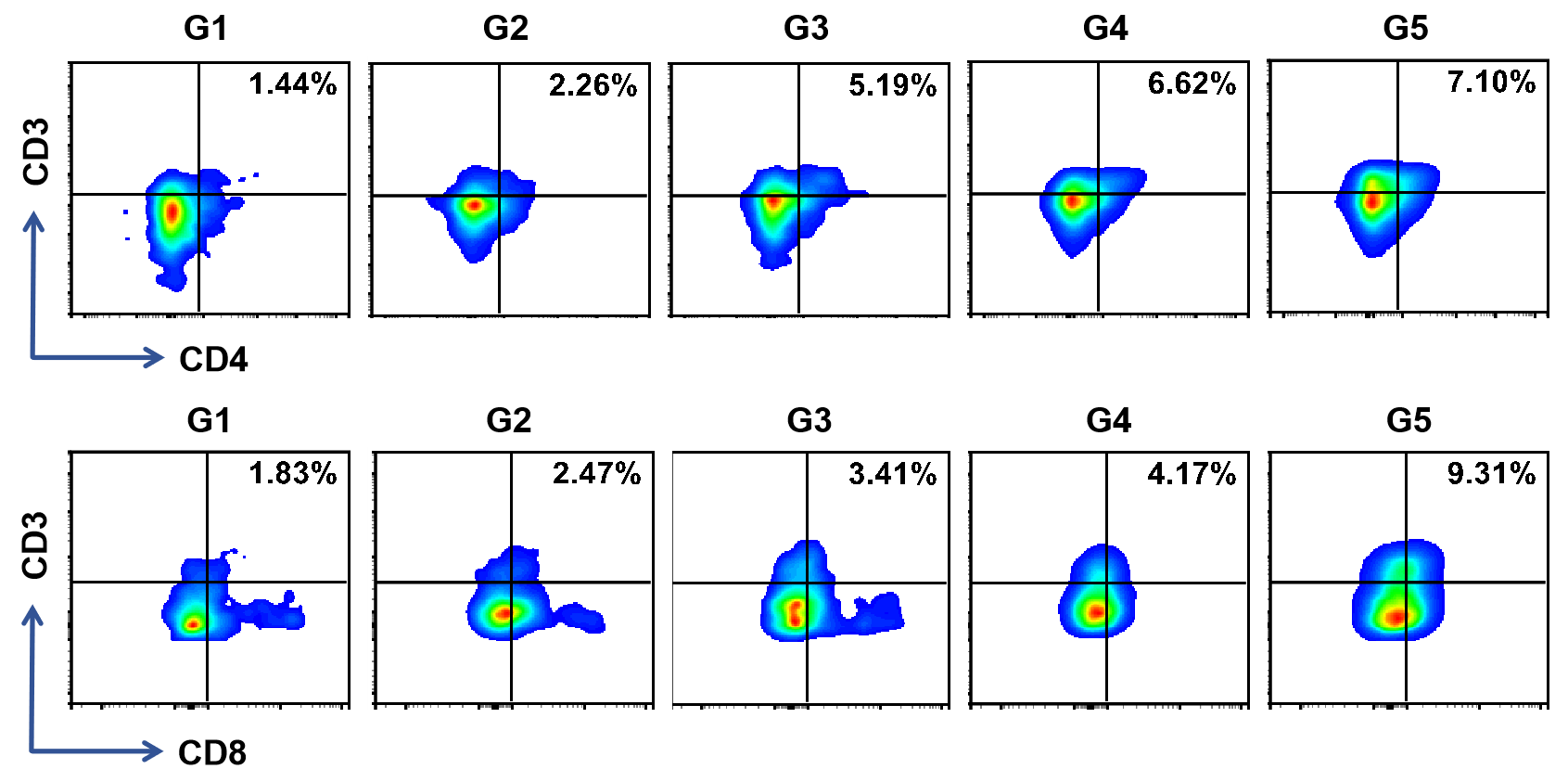


**Figure S28.** Flow cytometry analysis of CD4^+^ and CD8^+^ T cells in the spleen of mice.


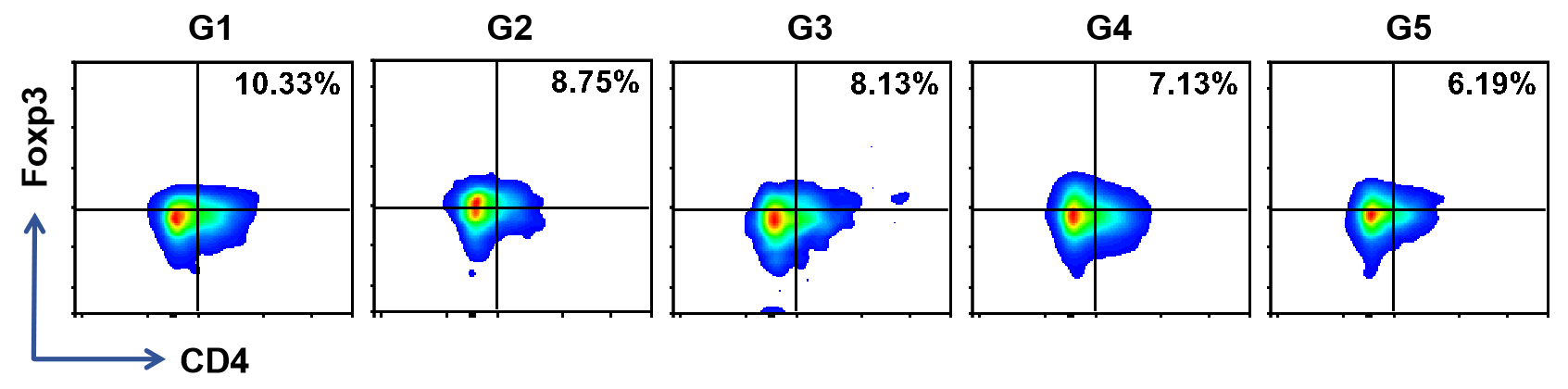


**Figure S29.** Flow cytometry analysis of Treg cells (CD4^+^Foxp3^+^) in the spleen of mice.


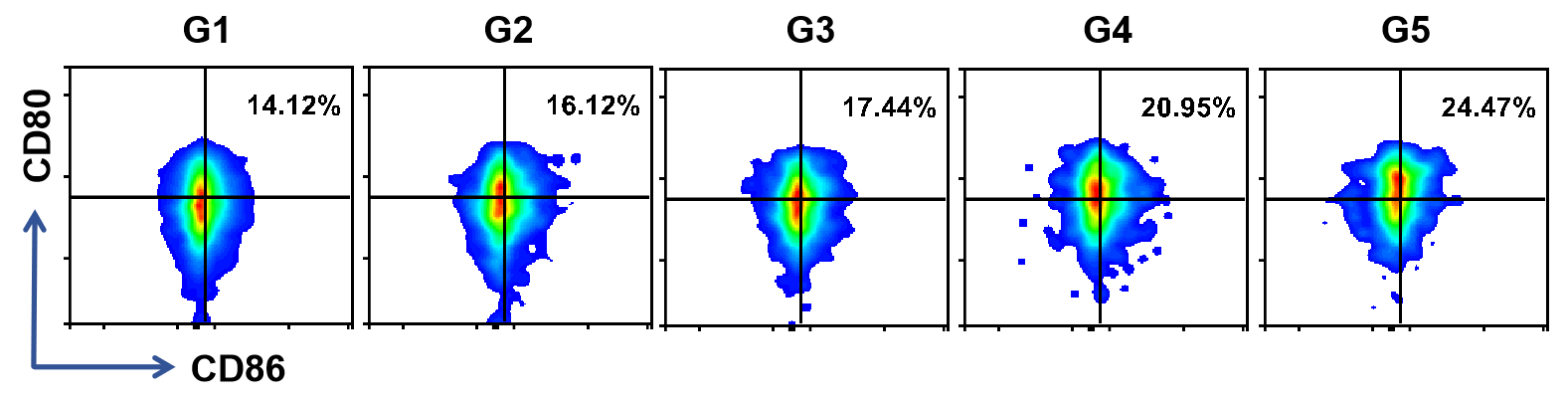


**Figure S30.** Flow cytometry analysis of mature DCs (CD80^+^CD86^+^) in peripheral blood.

**
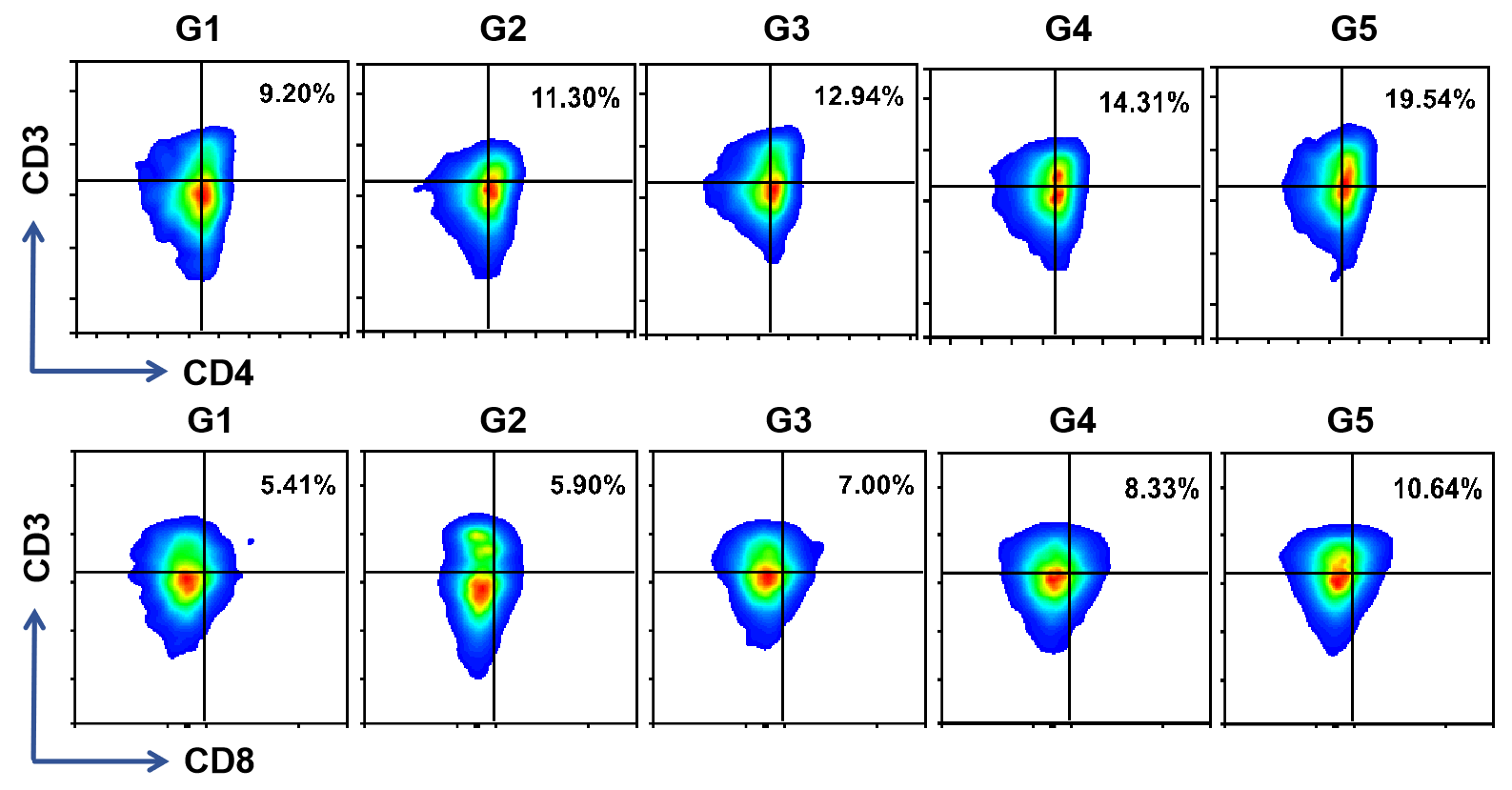
**

**Figure S31.** Flow cytometry analysis of CD4^+^ and CD8^+^ T lymphocyte populations in peripheral blood.

**
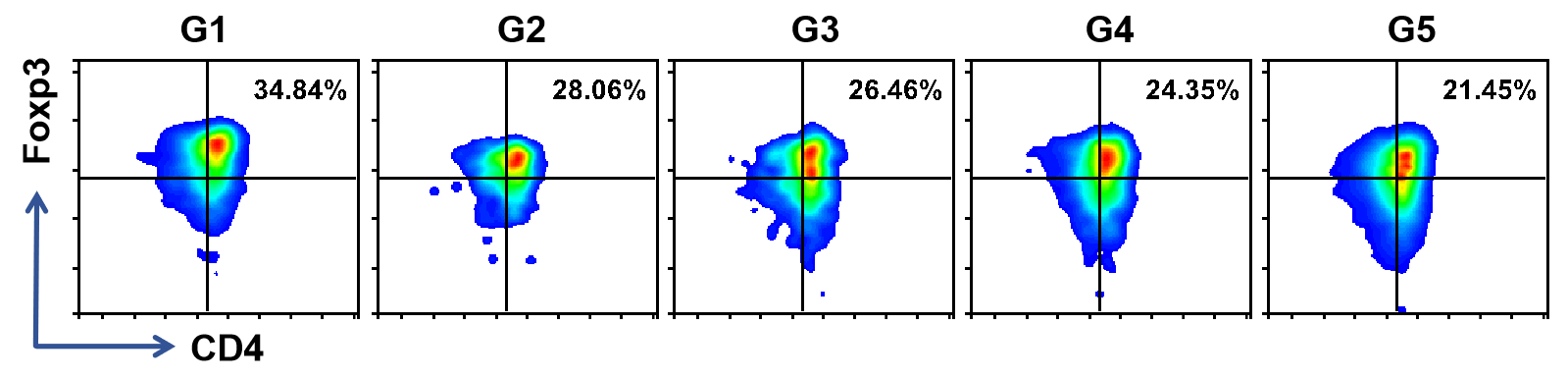
**

**Figure S32.** Flow cytometry analysis of Tregs (CD4^+^Foxp3^+^) in peripheral blood.
